# Transcriptional control of *Clostridium autoethanogenum* using CRISPRi

**DOI:** 10.1093/synbio/ysab008

**Published:** 2021-02-10

**Authors:** Nicholas Fackler, James Heffernan, Alex Juminaga, Damien Doser, Shilpa Nagaraju, R Axayacatl Gonzalez-Garcia, Séan D Simpson, Esteban Marcellin, Michael Köpke

**Affiliations:** 1 LanzaTech Inc, Skokie, IL 60077, USA; 2 Australian Institute for Bioengineering and Nanotechnology, ARC Centre of Excellence in Synthetic Biology & Metabolomics Australia, The University of Queensland, Brisbane, Queensland, 4072 Australia

**Keywords:** acetogen, *Clostridium autoethanogenum*, CRISPR/Cas9, CRISPRi, gas fermentation

## Abstract

Gas fermentation by *Clostridium autoethanogenum* is a commercial process for the sustainable biomanufacturing of fuels and valuable chemicals using abundant, low-cost C1 feedstocks (CO and CO_2_) from sources such as inedible biomass, unsorted and nonrecyclable municipal solid waste, and industrial emissions. Efforts toward pathway engineering and elucidation of gene function in this microbe have been limited by a lack of genetic tools to control gene expression and arduous genome engineering methods. To increase the pace of progress, here we developed an inducible CRISPR interference (CRISPRi) system for *C. autoethanogenum* and applied that system toward transcriptional repression of genes with ostensibly crucial functions in metabolism.

## Graphical Abstract

**Graphical Abstract:**
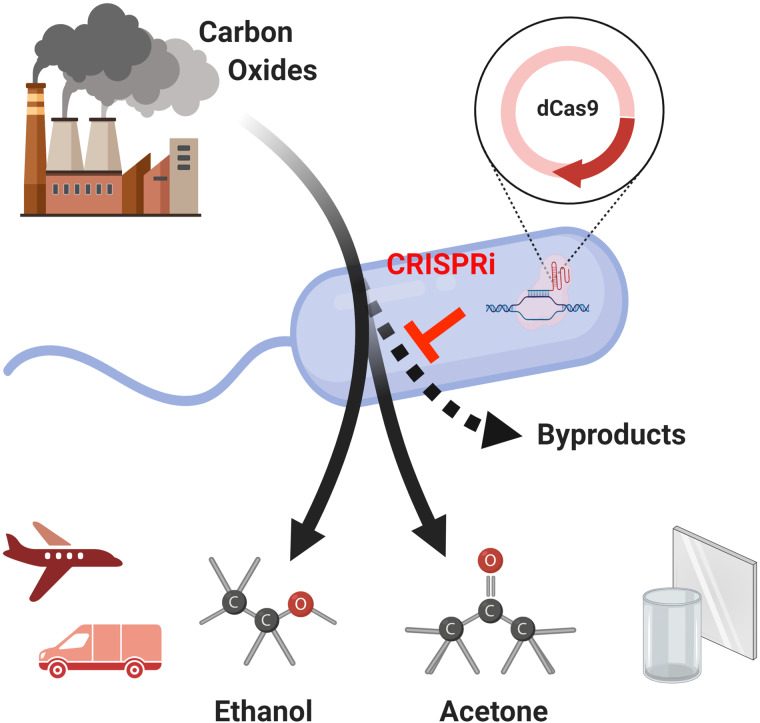

## 1. Introduction

The consequences of climate change continue to disrupt agricultural processes, water supplies, economies, and coastal communities and ecosystems. Emission of greenhouse gas (GHG), such as carbon dioxide (CO_2_), has been shown to have a growing influence on global warming (1). Despite Paris Agreement, signed by representatives of 194 nations, acknowledging the desperate need to reverse GHG emissions across the world, combustion of fossil fuels and the resulting release of (CO_2_) remains the largest and growing contributor of GHG (2, 3).

Gas fermentation by carbon fixing chemolithoautotrophic organisms, such as *Clostridium autoethanogenum*, offers an opportunity to recycle carbon and harness energy from synthesis gas (syngas) generated through gasification of organic biomass (such as agricultural waste, unsorted and nonrecyclable municipal solid waste or industrial waste) or industrial off-gases. Carbon recycled by *C. autoethanogenum* can be applied toward the production of transportation fuels, nutritionally valuable food additives for animal feed and chemicals (4–6). After a decade of research and progressive scale-up, the technology has been commercialized with the first plant operating successfully, producing fuel-grade ethanol, since 2018; additional units are currently under construction (7).

Synthetic biology and engineering approaches of gas fermenting organisms have yielded production of over 50 molecules of different chain length and chemistries (8). Some of these products including acetone (9, 10), isopropanol, 2,3-butanediol and 3-hydroxybutyrate (11) have been optimized in *C. autoethanogenum* for high titers, rates and selectivities. New tools such for genome engineering (12), creation of a high-throughput anaerobic biofoundry and cell-free prototyping (13) have increased rates of new strain development and scale-up. Despite this progress, challenges remain such as the relatively basic understanding of the regulatory networks of acetogens, low-throughput and laborious requirements of designing, cloning and screening genetic knockout (KO), and a surplus of uncharacterized, hypothetical genes in the genome.

Clustered Regularly Interspaced Short Palindromic Repeats (CRISPR) is a bacterial acquired immune system to combat phage infections that has revolutionized DNA engineering through recombinant expression in a wide variety of organisms (14–17). One such CRISPR nuclease, CRISPR-associated protein 9 (Cas9), from *Streptococcus pyogenes* is directed to a target sequence based on homology with a 20 nucleotide ‘guide’ that is often synthetically co-transcribed with chimeric crRNA-tracrRNA and referred to as a single guide RNA (sgRNA) (17). In the presence of the Protospacer Adjacent Motif (PAM) sequence ‘NGG’, Cas9 endonuclease cleaves proximal DNA creating a double-stranded break which is repaired by host DNA repair enzymes in the presence of homologous DNA fragments (17). CRISPR/Cas9-mediated genome modification has been shown in a diverse array of microbial systems including in a few Clostridia (18–22). We have previously demonstrated the use of Cas9 toward genome engineering in *C. autoethanogenum* (12). A yet to be addressed gap in the toolbox for *C. autoethanogenum* is the ability to generate genomic perturbations without burdensome, low-throughput laboratory methods involved in generating KO cell lines.

By introducing point mutations in the catalytic residues (D10A and H840A) of the gene encoding Cas9, the protein loses all DNA-cleaving capabilities but retains the ability to bind to DNA (17, 23). This enzymatically ‘dead’ Cas9 (dCas9) can be purposed toward disruption of gene expression by blocking proteins such as transcription factors and RNA polymerase (RNAP) from interacting with DNA through a process known as ‘CRISPR interference’ (CRISPRi) (23). When sgRNAs are localized upstream of the coding sequenced dCas9 blocks the initiation of transcription by occupying sequence motifs recognized by RNAP; whereas intragenic binding can prevent transcriptional elongation (23).

Here we show/report CRISPRi-mediated knockdown (KD) of gene expression in *C. autoethanogenum*. For the exemplification of our system, two genes were chosen that have previously been studied at the KO level, a promiscuous NADPH-dependent primary: secondary alcohol dehydrogenase (sADH; CAETHG_0553) which is capable of converting acetone to isopropanol and an alpha-acetolactate decarboxylase (BudA; CAETHG_2932) which is essential for the native production of 2,3-butanediol (12, 24). A recently identified *TetR* family transcriptional regulator (*TetR*; CAETHG_0459), associated with genes essential for autotrophic growth, was selected as a candidate that could be difficult to generate as a KO and may validate predicted mechanisms of transcriptional regulation of acetogens (25).

## 2. Materials and methods

### 2.1 Strain and cultivation

A derivative strain of *C. autoethanogenum* DSM10061 obtained from the German Collection of Microorganisms and Cell Cultures GmbH (DSMZ) was grown under strict anaerobic condition as described earlier using a synthetic gas blend, representative of waste gases from steel manufacturing, consisting of 50% CO, 10% H_2_ and 40% CO_2_ (Airgas) (26). Unless otherwise stated, all strains were handled using anaerobic techniques and media described earlier (26). Autotrophic growth experiments were completed in Schott Duran pressure bottles with rubber stoppers.

### 2.2 sgRNA annotation and scoring

sgRNA candidate sites were annotated using Geneious V9.1 (Biomatters) ‘CRISPR site finder’ tool which provides 20 bp target sequences adjacent to ‘-NGG’ PAM sites. On-target activity is predicted using previously published methods (27). This scoring algorithm analyses base features of the sgRNA, GC content and uses an experimentally determined predictive model to provide a score between 0 and 1 reflecting the expected activity level of the CRISPR target.

### 2.3 Assembly of CRISPR/Cas9 plasmids

Engineering of dCas9 from catalytically active Cas9 was accomplished using a modified quick-change PCR protocol (28). Active sites were mutagenized in sequential reactions, not simultaneous. Plasmids with unique sgRNA were generated using a modified quick-change PCR protocol wherein new sgRNA sequences were encoded at the overlapping ends of forward and reverse primers (28). Plasmids were amplified by PCR using primers (Supplementary Table S2), treated with DPN1 following NEB’s Time-Saver protocol and assembled using the ThermoFisher Seamless Plus kit.

### 2.4 Strain construction

*Clostridium autoethanogenum* strain construction was carried out as described earlier (26). Positive transformants were selected on agar plates containing 5 µg/ml clarithromycin and screened by PCR using primers 18001 and 18002 followed by next-generation sequencing (NGS) to confirm the presence of dCas9 and sgRNA sequence.

The list of all plasmids and oligonucleotides with sequences used in this work is listed in Supplementary Tables S1 and S2.

### 2.5 Strain screening

*Clostridium autoethanogenum* cultures were normalized to an inoculum of 0.02 in 10 ml of media and induced with 32 ng/ml of anhydrotetracycline. During screens for acetone to isopropanol conversion, 10 g/l of anaerobic acetone was added to the culture after 24 h of growth and a start-point sample was immediately taken. Strains continued to grow under the same conditions for 7 days at which time endpoint samples were taken. Ten milliliters broth samples were taken at 48 and 120 h for quantitative reverse transcription polymerase chain reaction (RT-qPCR) and pelleted by centrifugation at 4°C for 5 min, flash frozen in liquid nitrogen and stored at -80°C for omics analysis.

### 2.6 NGS quality control

Purified plasmids and PCR products were sequenced using the Illumina MiSeq. Geneious software v9.1 was used to align Fastq files to reference sequences for variant analysis.

### 2.7 Quantitative reverse transcription polymerase chain reaction

KD of gene expression was confirmed using two-step RT-qPCR. RNA extractions of harvested, flash-frozen cell pellets were performed using RiboPure™ RNA Purification Kit (ThermoFisher Scientific). cDNA was synthesized using GoScript™ Reverse Transcriptase (Promega). Expression was quantified by qPCR of cDNA compared to a gBlock^®^ (Integrated DNA Technologies, IDT) reference containing genes of interest ± 50 bp (Supplementary Table S2). Cycle threshold (C_t_) values were found using the synthesized cDNA, QX200™ ddPCR™ EvaGreen Supermix (Bio-Rad), and primers (Supplementary Table S2) from IDT in opaque-96-well plates (Bio-Rad) with a CFX96 Real-Time System (Bio-Rad). All experimental steps were completed following the manufacturer’s protocols. CAETHG_0459 C_t_ values and copy number were normalized within biological replicates by all of *C. autoethanogenum’*s 16S rRNA genes (Supplementary Table S3), as a control for variability in aspects such as; efficiencies of RNA extraction and reverse transcription steps (29). Furthermore, 16S genes were chosen to normalize expression as their transcriptional regulation was not found to be ‘controlled’ by CAETHG_0459 (25). Choosing expression normalization genes that were not regulated by TetR was found to be a key aspect for accurately assessing KD effectiveness of a transcriptional regulator such as TetR. CAETHG_0533-Guide-4 samples were controls for calculating change in expression (relative expression) of CAETHG_0459 after KD.

### 2.8 RT-qPCR analysis

CFX Manager™ Software 3.1 (Bio-Rad) calculated gene copy numbers from samples’ C_t_ values and copy number/C_t_ standard curves (Supplementary Tables S4 and S5). Changes in expression were quantified using both copy number and adjusted C_t_ values (Supplementary Table S6). E^−ΔΔCt^ and Pfaffl methods were used to calculate relative expression from adjusted C_t_ values (Supplementary Table S7) (30). Full description of detailed relative expression analysis is shown in Supplementary Text, summarized in Supplementary Table S11. E^−ΔCt^ and copy number ratio (i.e. C#_TetR_/C#_16S_) data were used to calculate relative expression by either fitting to a normal distribution after logarithmic transformation or fit directly to a negative binomial distribution. A log-transform was used to correct for right skew, and can be an important step before statistical analysis (29, 31).

### 2.9 Western blots

Cultures were harvested during log-phase growth. Seven microliters 4X sample buffer [22% (vol/vol) 0.5 mM Tris-HCL pH 6.8, 43% (vol/vol) 100% glycerol (Sigma-Aldrich), 0.07% bromphenol blue (Sigma-Aldrich) and 34% (vol/vol) 10% SDS page buffer (Bio-Rad)], 23 µl culture, and 1 µl beta-mercaptoethanol (Sigma-Aldrich) were mixed and immediately lysed by heat treatment at 100°C for 5 min. Lysed samples and a precision plus protein standard (Bio-Rad) were loaded to a 7.5% Mini-ProTEAN TGX Gel (Bio-Rad). The gel was subjected to electrophoresis at 120 V for 1 h. The gel was sandwiched to a Trans-Blot nitrocellulose membrane (Bio-Rad), following the manufacturer’s protocol for the Trans-Blot Turbo Transfer system (Bio-Rad), and proteins were transferred to the membrane via electrophoresis at 25 V 2.5 A for 10 min. The membrane was treated with 30 ml freshly prepared blocking buffer [5% (wt/vol) BSA (Sigma-Aldrich), 0.1% (vol/vol) Tween20 (Sigma-Aldrich) in 1X PBS] for 1 h at room temperature in a reagent trough with mild agitation on an orbital shaker. Three 30 ml washes of 1X TBST (50 mM Tris-HCl, 150 mM NaCl, 0.1% [vol/vol] Tween [pH 7.5], in molecular grade water) were applied to membrane for 5 min at RT with mild agitation. Guide-iT Cas9 Polyclonal Antibody (TaKaRa) was diluted 1:1000 in unused blocking buffer and 30 ml was used to treat the membrane for 2 h at RT with mild agitation. Three additional 30 ml washes of TBST were applied to the membrane for 5 min at RT with mild agitation. A secondary antibody, Goat anti-Mouse-IgG-HRP (Invitrogen), was diluted 1:5000 in unused blocking buffer and 30 ml was used to treat the membrane for 1 h at RT with mild agitation. Three 30 ml washes of TBST were applied to the membrane for 5 min at RT with mild agitation. Fresh color reaction solution [2.5 mM 3,3ʹ-diaminobenzidine, 0.04% (vol/vol) H _2_O_2_ in 1X TBS (50 mM Tris-HCl, 150 mM NaCl, in molecular grade water)] was prepared. The membrane was transferred to a clean tray and exposed to the color reaction solution for 5 min then the reaction was stopped with deionized H_2_O. The membrane was air dried for 30 min before an image was captured.

### 2.10 Analytics

2,3-butanediol, acetone and isopropanol concentrations were measured as described earlier (11). OD_600nm_ was used for biomass measurements.

### 2.11 Availability of the materials

Materials are available upon reasonable request and under material transfer agreement, but strains may require a license.

## 3. Results

Initial proof of concept for the CRISPR/Cas9 system in *C. autoethanogenum* employed a dual plasmid system (12). To streamline the process, we recoded the D10A and H840A of the Cas9 system and simplify deployment as a single-plasmid construct (pCRISPRi) employing both a catalytically inactive Cas9 enzyme (dCas9) driven by a tetracycline-inducible promoter (IPL-12) and a constitutively expressed sgRNA to enable the control of gene transcription with CRISPRi.

**Figure 1.**
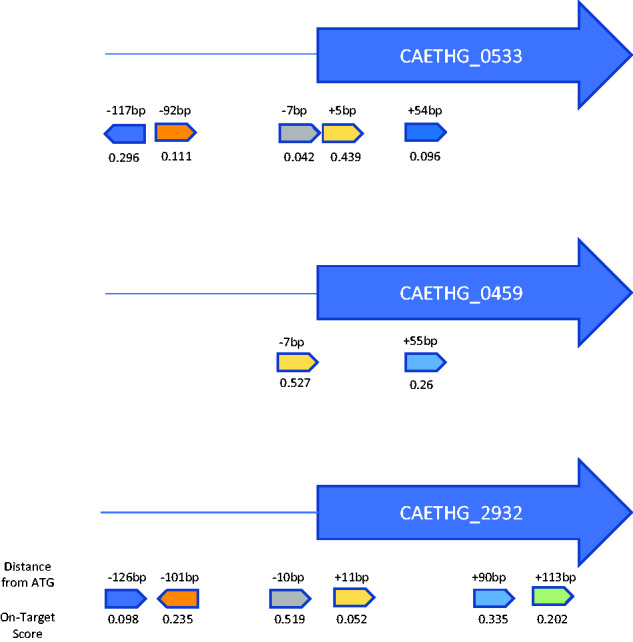
sgRNA targets for sADH (CAETHG_0553), TetR (CAETHG_0459 and BudA (CAETHG_2932). Distance in base pairs (bp) between the first bp of the start codon and the PAM site is listed on the top of the guide. On-target activity scores are provided below each guide. Guides IDs numerically assigned with the most 5ʹ as ‘CAETHG_XXX-Guide-1’.

Toward characterization of a spectrum of CRISPRi KD phenotypes in *C. autoethanogenum*, multiple plasmids with varying sgRNA sequences were generated for each target gene (Figure 1). Plasmids harboring unique sgRNA sequences were transformed into *C. autoethanogenum* and verified via PCR followed by NGS. Biological triplicates were batch cultured in selective media and induced with 32 ng/ml anhydrotetracycline; however, western blot data show that IPL-12 drives some dCas9 production without induction (Supplementary Figure S1).

In this study, we applied CRISPRi to KD expression of three genes of interest—a secondary alcohol dehydrogenase (sADH), alpha-acetolactate decarboxylase (BudA) and a TetR family transcriptional regulator (TetR). sADH and BudA are pivotal for control of carbon-flux in *C. autoethanogenum* and perturbations in their function can be phenotypically observed through measurements of the metabolites isopropanol and 2,3-butanediol, respectively. Compared to a negative control, strains with single sgRNA CRISPRi KD of sADH decreased conversion of acetone to isopropanol up to 95.7%; similarly, sgRNA KD of BudA led to a decrease of 2,3-butanediol production up to 100%. The TetR KD led to no conclusive changes in growth or metabolite production (phenomics); therefore, validation required analysis of mRNA levels which was achieved through RT-qPCR and demonstrated a 30-fold reduction of TetR expression. In total, these results demonstrate the utility of CRISPRi for reducing gene expression in *C. autoethanogenum*.

### 3.1 KD of 2,3-butanediol formation

**Figure 2.**
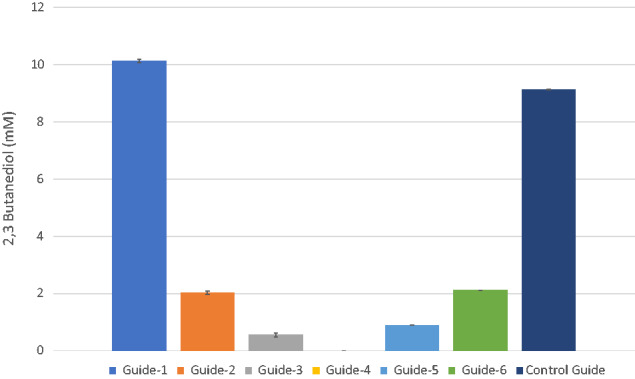
Production of 2,3-butanediol by CAETHG_2932 KD cultures. Data shown are from endpoint samples taken after cultures had sustained lag-phase. CAETGH_0533-Guide-4 was used as a control guide. Data represented as bar graphs are average of biological replicates (*N* = 3), where error bars represent standard deviation.

Directing CRISPRi to −101 - +113 bp region surrounding the start codon of BudA led, as expected, to reduced 2,3-butanediol formation. With exception of CAETHG_2932-Guide-1, the most distant sgRNA (−126 bp), KD was successful for all selected guides. 2,3-butanediol production was completely abolished by CAETHG_2932-Guide-4 (Figure 2).

### 3.2 KD of acetone to isopropanol conversion

**Figure 3.**
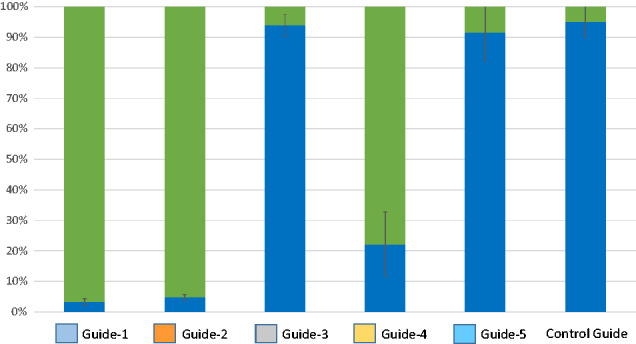
Conversion of acetone (green) to isopropanol (blue) by CAETHG_0533 KD cultures as demonstrated in a percentage of acetone to isopropanol at endpoint samples taken after cultures had sustained lag-phase. CAETHG_2932-Guide-2 was used as a control. Data represented as bar graphs are average of biological replicates (*N* = 3), where error bars represent standard deviation of isopropanol production.

Conversion of acetone to isopropanol was studied through spiking experiments. The control culture is readily able to reduce acetone to isopropanol, whereas strains with more functional sADH-targeting sgRNAs (CAETHG_0533-Guide-1, -2, -4) showed decreased conversion of acetone to isopropanol with some strains converting <5% of acetone to isopropanol (Figure 3). Here, the sgRNA with the lowest predicted on-target activity scores (CAETHG_0533-Guide-3 and Guide-5) were least effective.

### 3.3 Knockdown of TetR

**Figure 4.**
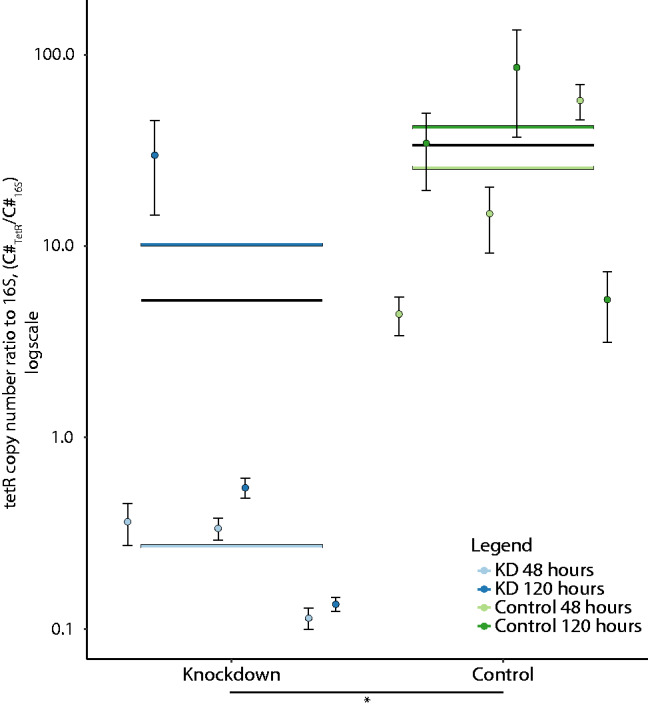
Analysis of TetR (CAETHG_0459) KD effect on expression using RT-qPCR. Copy number (C#) ratio of TetR to 16S from KD (guide-2 (+79), blue) and negative control (CAETHG_0533 guide-4, green). Data represented as points are average of technical replicates, where error bars represent standard deviation. The points are grouped by biological replicate (*N* = 3) and split into 48 and 120 h sampling points (light color/left and dark color/right, respectively). Bars represent means of data and are associated to their respective points if colored, while black bars are associated to KD and control (i.e. both timepoints). The difference between the means of KD and control samples was shown to be statistically significant using various methods, represented as *P* < 0.05 here (see Supplementary Text for various methods’ *P*-values).

Although not unexpected in an ‘AT’ rich genome like that of *C. autoethanogenum*, the ‘NGG’ PAM requirement of Cas9 was quite restrictive for TetR sgRNA design. Only a single candidate PAM site was located within −150 bases of the start codon, thus fewer sgRNAs were constructed targeting CAETHG_0459. Reduced expression of TetR could not be discriminated through endpoint analysis of phenotype, so strains were harvested for analysis at various stages of growth and RT-qPCR was used to quantify the transcriptional effect of KD on TetR expression. Comparison of KD and negative control samples showed differential expression of TetR (Figure 4), with E^−ΔΔCt^ and Pfaffl calculation methods finding the relative expression between samples to be 0.036 and 0.034 (∼30-fold decrease), respectively (Supplementary Table S7). To confirm significance of the change in relative expression, a more comprehensive analysis of relative expression was performed using similar methods (Table 1, Supplementary Text).

**Table 1.** Calculation of relative expression and comparison of calculation methods

**Method**	**Relative expression**			**Prediction using model means (E^-ΔCt^, C#_TetR_/C#_16S_)**	
	µ	**LL** ^a^	**UL** ^a^	KD	Control
log(E^ΔCt^)^b^	0.031	0.002	0.421	0.37	11.97
Outlier removed	(0.015)	(0.002)	(0.089)	(0.17)	(11.81)
log(C#_TetR_/C#_16S_)^b^	0.028	0.002	0.409	0.56	19.69
Outlier removed	(0.013)	(0.002)	(0.082)	(0.26)	(19.39)
lm(log(C#_TetR_/C#_16S_))^c^	0.028	0.003	0.249	0.56	19.68
Outlier removed	(0.013)	(0.003)	(0.052)	(0.25)	(19.68)
glm.nb(C#_TetR_/C#_16S_)^c^	0.155	0.027	0.883	4.99	32.29
Outlier removed	(0.009)	(0.004)	(0.025)	(0.30)	(32.29)

^a^ Lower and upper limit of 95% confidence intervals.

^b^ Analysis performed with SigmaPlot 13.0 ANCOVA analysis, using the Holm–Sidak method.

^c^ Analysis performed in R using linear models (lm from package stats) (34) and generalized linear models, with negative binomial (glm.nb from package MASS) (35) models, where Confint from car package (36) found the CIs. Values in brackets are results when removing the potential outlier from model data set (KD biological replicate 1, day 5). For reference, arithmetic means of E^−ΔCt^ are 3.12 and 20.05 for KD and control samples, respectively; and of C#_TetR_/C#_16S_ are 5.22 and 33.69 for KD and control samples, respectively. See Supplementary Text for full details of analyses.

It is notable that no phenomic change was observable although there was a large change in relative expression of TetR. This shows there is further complexity to the condition-specific transcriptional architecture of *C. autoethanogenum* (32) yet to be uncovered. Additionally, it is a reasonable to expect that over-expression of TetR will have a phenomic effect similar to KD (25). A mechanistic description of TetR’s role as a sigma factor would particularly enhance the understanding of *C. autoethanogenum*’s transcriptional architecture.

Mean relative expression of KD samples fell between 0.029 and 0.155 from various analytical methods demonstrating a ∼ 6.5- to 35.7-fold decrease from control strains (Table 1). The sampling timepoint did not have a significant effect on the relative expression. The right skew of the data can be visualized in Figure 4 as increasing standard deviation with copy number ratio (see Supplementary Text). Data from KD, bioreplicate 1, day 5 sample were identified as a potential outlier by lm and glm.nb models (Supplementary Text), and this data value had a large impact on relative expression analysis (Table 1). Removal of the outlier changed the mean relative expression to a 66.7- to 111.1-fold decrease. Removal of the outlier was shown to reduce the relative error between calculation methods (i.e. 35.7/6.5 = 5.5 versus 111.1/66.7 = 1.7).

## 4. Conclusion

We found that sgRNA targeting regions proximal (∼100 bp) to the start codon with were most effective at generating strong KD phenotypes in *C. autoethanogenum*. The effective targeting region is likely to not be static across the genome as transcriptional start sites differ based on the upstream sequence and sigma factor system involved in expression (33). All guides with on-target scores above 0.1 elicited some degree of KD. The variation in effectiveness enables tunability in the system where certain sgRNA allow for reduction without eliminating gene expression, which is useful for studying essential genes where generating a KO cell line is difficult or impossible. As reported elsewhere, some sgRNA appeared nonfunctional (37, 38). We suspect this to be caused by structural difficulties with the gRNA accessing the genomic DNA, off-target binding that we are not experimentally observing, or incorrect assumptions about the range enabling RNAP interference. It is possible that a perfect scoring system alongside a well-defined transcriptional start site one could predictably elicit strong KD of gene expression; however, at this time, it is impossible to be sure if sgRNAs are effective without testing. Availability of PAM sites was restrictive for only one of our target genes; a future improvement could be to engineer a system with an ‘AT’ rich PAM requirement such as the ‘TTTV’ PAM of some Cpf1 (CAS14a1) variants (39).

Strains containing CAETHG_2932-Guide-2 (BudA) and CAETHG_0533-Guide-4 (sADH) were re-purposed as control plasmids in counter-experiments as they contain an active dCas9 system targeting a gene not to be involved in the pathway of interest; functional systems were a preferred control as production of dCas9 has been observed to elicit fitness burdens on bacterial strains (40). The cause of the burden has not been fully characterized. Cas9, and thus dCas9, has been shown to function by first actively ‘scanning’ the genome through opening double-stranded DNA in search for the PAM motif, and then checking the complementarity of the sgRNA sequence to the bona fide target site (37). It is possible that by opening and occupying the DNA dCas9 is causing a burden through random prevention of cell directed protein–DNA interaction. It is more likely that nonspecific binding of dCas9 toward off-target sequences, which has been demonstrated in *E. coli* with as little as 9 nt of match, leads to a decrease in fitness (40). Some groups choose to use a dCas9 without a ‘functional’ sgRNA as a control, but it is extremely difficult to be certain that a sgRNA is ‘nonfunctional’ as off-target effects can be observed, so we chose to use a functional guide that targeted another, characterized system.

We extended the molecular toolbox for the model acetogen *C. autoethanogenum*, by developing and demonstrating a CRISPRi system for the repression of gene expression for genes involved in metabolism and transcriptional regulation. This tool will be useful for studying gene function of under-characterized genes, metabolic flux, and essential genes in this industrially relevant class of organisms, as well as other clostridial systems. The ease of sgRNA swapping and single-plasmid nature of this tool allows for a shortened design-build-test analyze cycles compared to generating KO strains, targeting of essential genes for which loss of function is not tolerable, and construction of genome-wide KD libraries with minimal cloning effort. The ability to generate restricted KD without eliciting full loss of function allows for expression of essential genes to be engineered with unprecedented ease for clostridia and acetogens. Application of this tool as a CRISPRi library will open opportunities for functional and fitness assays that can elucidate genomic landscapes, especially those of acetogens which contain numerous hypothetical genes, long-noncoding RNAs, and unknown regulatory controls at a genome-wide level. Our CRISPRi system should be adaptable with other recent CRISPR-based genome engineering advances such as multiplexing KDs with nonrepetitive extra-long sgRNA arrays, fitness screening with genome-wide KD libraries, base editing via deamination and CRISPR activation (CRISPRa) and could serve to enhance the molecular tool box of several clostridial and acetogenic species (41–43).

## SUPPLEMENTARY DATA

Supplementary Data are available at SYNBIO Online.

## Supplementary Material

ysab008_Supplementary_Data

## Data Availability

The authors confirm that the data supporting the findings of this study are available within the article and/or its supplementary materials.
