# Supplementary material for: Transcriptional control of Clostridium autoethanogenum using CRISPRi
Source: Synth Biol (Oxf). 2021 Feb 10;6(1):ysab008. doi: 10.1093/synbio/ysab008 (PMC8062849; doi:10.1093/synbio/ysab008)
Supplement: ysab008_Supplementary_Data [file ysab008_supplementary_data.zip › Supplementary Text.pdf]

Supplementary Material For

Transcriptional Control of *Clostridium autoethanogenum* using CRISPRi

Nick Fackler[1]*, James Heffernan[2], Alex Juminaga[1], Damien Doser[1], Shilpa Nagaraju[1], R. Axayacatl Gonzalez-Garcia[2], Sean D. Simpson[1], Esteban Marcellin[2], Michael Köpke[1]*

[1] LanzaTech Inc., 8045 Lamon Ave, Suite 400, Skokie, IL 60077, USA
[2] Australian Institute for Bioengineering and Nanotechnology, The University of Queensland, Brisbane, QLD 4072, Australia

*Corresponding authors: Nick Fackler (nick.Fackler@lanzatech.com), Michael Köpke (michael.koepke@lanzatech.com)

Content:

Supplementary Figure 1: Western blot data demonstrating Cas9 production by pCRISPRi....................2

SigmaPlot analysis of qPCR experiments

One Way Analysis of Covariance – Dependent Variable: 2^dCt 3
One Way Analysis of Covariance – Dependent Variable: log(2^dCt) 4
One Way Analysis of Variance – Dependent Variable: log(2^dCt) 5
One Way Analysis of Covariance - Dependent Variable: dCt 6
One Way Analysis of Covariance Dependent Variable: dCt_KD 7
One Way Analysis of Covariance – Dependent Variable: C#/C# 8
One Way Analysis of Covariance – Dependent Variable: log(C#/C#) 9
One Way Analysis of Covariance – Dependent Variable: log(2^dCt)_KD 10
One Way Analysis of Covariance – Dependent Variable: log(C#/C#)_KD 11

Supplementary file describing analysis of qPCR data in R *(page numbering restarts)*

Summary 12
Loading package libraries and data 13
Manipulating data for various uses....................15
Graphing data 17
Assessing normality 19
Generalised linear model (Poisson) 26
Generalised linear model (negative binomial)....................30
Negative binomial model without outlier 35
Linear models of log transformed copy number ratio 41
Calculating 95% CIs for comparison 44
Validating and comparing models with 'performance'....................56
Analysis references....................58

(SigmaPlot and R documents joined manually)

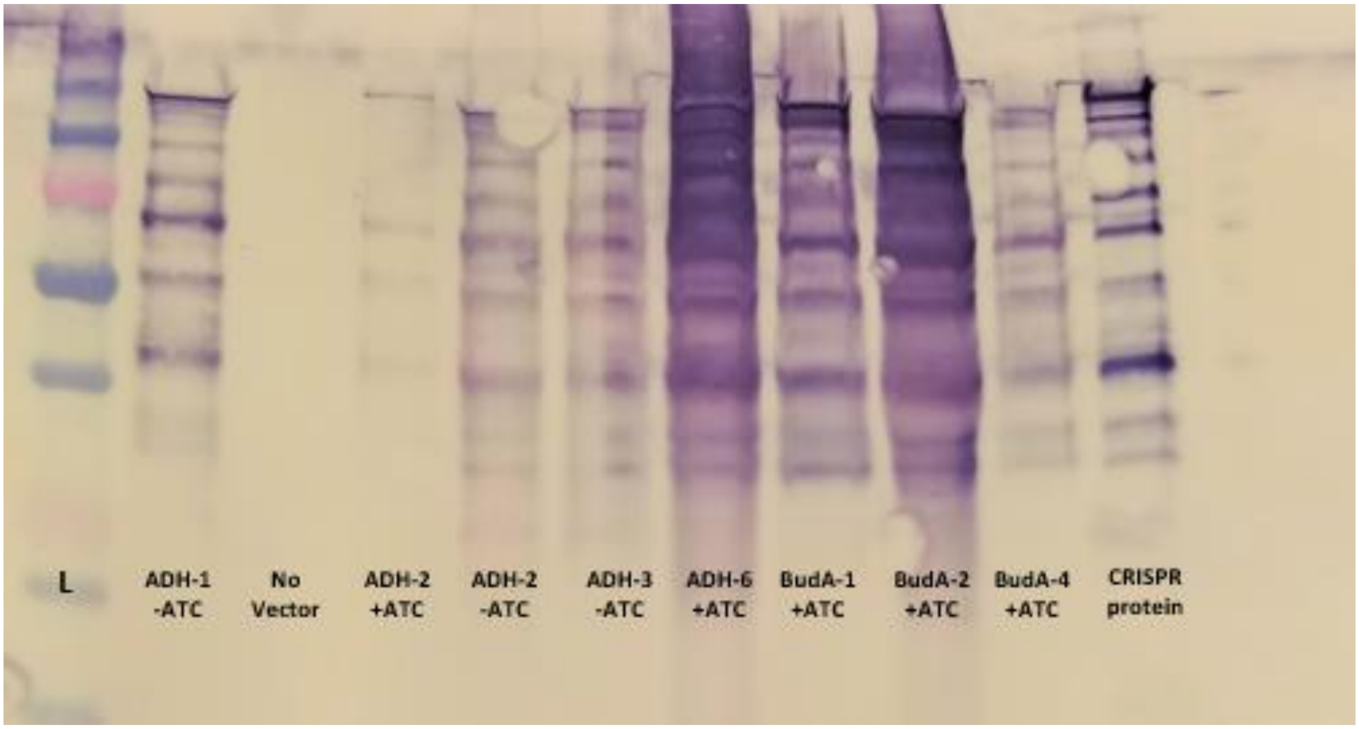

Supplementary Figure 1: Western blot data demonstrating Cas9 production by pCRISPRi. Some cultures were split into induced media containing 32 ng/mL anhydrotetracycline (+ATC) and uninduced media (-ATC) to demonstrate protein production. 50 ng purified Cas9 protein from Zageno (K008) was used as a positive control and *C. autoethanogenum* without a vector was used as a negative control.

SigmaPlot analysis of qPCR experiments

Table 1: "Data 1" for SigmaPlot analysis

Day	Sample	Bioreplicate	dCt	2^dCt	log(2^dCt)	C#/C#	log(C#/C#)
2	KD	BR1	2.716	0.236	-1.444	0.363	-1.014
2	KD	BR2	2.726	0.235	-1.449	0.335	-1.094
2	KD	BR3	4.769	0.0793	-2.535	0.117	-2.174
2	N	BR1	-1.884	2.722	1.002	4.410	1.484
2	N	BR2	-4.192	9.281	2.228	14.755	2.692
2	N	BR3	-6.696	35.140	3.559	57.569	4.053
**5**	**KD**	**BR1**	**-5.410**	**17.740**	**2.876**	**29.834**	**3.396**
5	KD	BR2	1.911	0.362	-1.016	0.546	-0.606
5	KD	BR3	4.545	0.089	-2.416	0.134	-2.008
5	N	BR1	-5.738	21.113	3.045	34.426	3.539
5	N	BR2	-7.316	48.851	3.889	85.749	4.451
5	N	BR3	-2.189	3.201	1.163	5.251	1.658

Potential outlier bolded, indicated by _KD in SigmaPlot analysis.
See Supplementary file # for full calculations of "Data 1".

One Way Analysis of Covariance – Dependent Variable: 2^dCt
Data source: Data 1

Group Name	N	Missing	Mean	Std Dev	SEM
KD	6	0	3.124	7.161	2.924
N	6	0	20.052	18.754	7.656
Total	12	0	11.588	16.166	4.667

Normality Test (Shapiro-Wilk): Passed (P = 0.614)
Equal Variance Test (Levene): Failed (P < 0.050)
Equal Slopes Test: Passed (P =0.878)

Analysis of Variance for the Interaction Model:

Source of Variation	DF	SS	MS	F	P
Sample	1	86.978	86.978	0.376	0.557
Day	1	158.866	158.866	0.687	0.431
Sample x Day	1	5.852	5.852	0.0253	0.878
Residual	8	1850.171	231.271	--	--
Total	11	2874.564	261.324	--	--

The effect of the different treatment groups does not depend upon the value of covariate Day, averaging over the values of the remaining covariates. There is not a significant interaction between the factor Sample and the covariate Day (P = 0.878).
There are no significant interactions between the factor and the covariates. The equal slopes assumption passes and the equal slopes model is analyzed below.

Analysis of Equal Slopes Model:
R = 0.595 Rsqr = 0.354 Adj Rsqr = 0.211
Analysis of Variance for the Equal Slopes Model:

Source of Variation	DF	SS	MS	F	P
Sample	1	859.674	859.674	4.169	0.072
Day	1	158.866	158.866	0.770	0.403
Residual	9	1856.024	206.225	--	--
Total	11	2874.564	261.324	--	--

The differences among the adjusted means of the treatment groups are not great enough to exclude the possibility that the differences are only due to random sample variability. There is not a significant difference between the adjusted means (P = 0.072).
The coefficient of covariate Day in the equal slopes regression model is not significantly different from zero (P = 0.403).
There is no evidence that the covariate affects the values of dependent variable; you may want to consider removing it from the regression model.
No covariate significantly contributes to the values of the dependent variable. You may want to consider a single factor ANOVA design for your study.

Adjusted Means of the Groups:

Group Name	Adjusted Mean	Std. Error	95%Conf-L	95%Conf-U
KD	3.124	5.863	-10.139	16.386
N	20.052	5.863	6.789	33.314

The adjusted means are the predicted values of the dependent variable 2^dCt for each group where each covariate variable is evaluated at the average of its data values.
Regression Equations for the Equal Slopes Model:
There is no significant difference in the intercepts of the dependent variable for these equations since there is no significant difference in the adjusted means of the factor groups (P = 0.072).
Group: KD
2^dCt = -5.366 + (2.426 * Day)
Group: N
2^dCt = 11.562 + (2.426 * Day)

One Way Analysis of Covariance – Dependent Variable: log(2^dCt)
Data source: Data 1

Group Name	N	Missing	Mean	Std Dev	SEM
KD	6	0	-0.997	1.989	0.812
N	6	0	2.482	1.221	0.499
Total	12	0	0.742	2.404	0.694

Normality Test (Shapiro-Wilk): Passed (P = 0.410)
Equal Variance Test (Levene): Passed (P = 0.708)
Equal Slopes Test: Passed (P = 0.561)
Analysis of Variance for the Interaction Model:

Source of Variation	DF	SS	MS	F	P
Sample	1	11.010	11.010	3.830	0.086
Day	1	3.188	3.188	1.109	0.323
Sample x Day	1	1.056	1.056	0.367	0.561
Residual	8	22.995	2.874	--	--
Total	11	63.551	5.777	--	--

The effect of the different treatment groups does not depend upon the value of covariate Day, averaging over the values of the remaining covariates. There is not a significant interaction between the factor Sample and the covariate Day (P = 0.561).
There are no significant interactions between the factor and the covariates. The equal slopes assumption passes and the equal slopes model is analyzed below.
Analysis of Equal Slopes Model:
R = 0.788 Rsqr = 0.622 Adj Rsqr = 0.537
Analysis of Variance for the Equal Slopes Model:

Source of Variation	DF	SS	MS	F	P
Sample	1	36.313	36.313	13.589	0.005
Day	1	3.188	3.188	1.193	0.303
Residual	9	24.050	2.672	--	--
Total	11	63.551	5.777	--	--

The differences in the adjusted means among the treatment groups are greater than would be expected by chance; there is a statistically significant difference (P = 0.005). To isolate which group(s) differ most from the others use a multiple comparison procedure. The adjusted means and their statistics are given in the table below.
The coefficient of covariate Day in the equal slopes regression model is not significantly different from zero (P = 0.303). There is no evidence that the covariate affects the values of dependent variable; you may want to consider removing it from the regression model.
No covariate significantly contributes to the values of the dependent variable. You may want to consider a single factor ANOVA design for your study.
Adjusted Means of the Groups:

Group Name	Adjusted Mean	Std. Error	95%Conf-L	95%Conf-U
KD	-0.997	0.667	-2.507	0.512
N	2.482	0.667	0.972	3.992

The adjusted means are the predicted values of the dependent variable log(2^dCt) for each group where each covariate variable is evaluated at the average of its data values.
All Pairwise Multiple Comparison Procedures (Holm-Sidak method):
Comparisons for factor: **Sample**

Comparison	Diff of Means	t	P	P<0.050
N vs. KD	3.479	3.686	0.005	Yes

Regression Equations for the Equal Slopes Model:
There is a significant difference in the intercepts of the dependent variable for these equations since there is a significant difference in the adjusted means of the factor groups (P = 0.005).
Group: KD
log(2^dCt) = -2.200 + (0.344 * Day)
Group: N
log(2^dCt) = 1.279 + (0.344 * Day)

One Way Analysis of Variance – Dependent Variable: log(2^dCt)

Normality Test (Shapiro-Wilk): Passed (P = 0.056)
Equal Variance Test (Brown-Forsythe): Passed (P = 0.869)

Group Name	N	Missing	Mean	Std Dev	SEM
KD	6	0	-0.997	1.989	0.812
N	6	0	2.482	1.221	0.499

Source of Variation	DF	SS	MS	F	P
Between Groups	1	36.313	36.313	13.331	0.004
Residual	10	27.238	2.724		
Total	11	63.551			

The differences in the mean values among the treatment groups are greater than would be expected by chance; there is a statistically significant difference (P = 0.004).
Power of performed test with alpha = 0.050: 0.885

Multiple Comparisons versus Control Group (Holm-Sidak method):
Overall significance level = 0.05

Comparisons for factor: **Sample**

Comparison	Diff of Means	t	P	P<0.050
N vs. KD	3.479	3.651	0.004	Yes

NOTE: ANOVA and ANCOVA analysis produces the same result due to the insignificance of the 'Day' variable. ANCOVA analyses were used as the default analysis moving forward to ensure that 'Day' remained an insignificant variable.

One Way Analysis of Covariance - Dependent Variable: dCt
Data source: Data 1

Group Name	N	Missing	Mean	Std Dev	SEM
KD	6	0	1.876	3.742	1.528
N	6	0	-4.669	2.297	0.938
Total	12	0	-1.397	4.522	1.305

Normality Test (Shapiro-Wilk): Passed (P = 0.410)
Equal Variance Test (Levene): Passed (P = 0.708)
Equal Slopes Test: Passed (P = 0.561)
Analysis of Variance for the Interaction Model:

Source of Variation	DF	SS	MS	F	P
Sample	1	38.962	38.962	3.830	0.086
Day	1	11.283	11.283	1.109	0.323
Sample x Day	1	3.734	3.734	0.367	0.561
Residual	8	81.383	10.173	--	--
Total	11	224.925	20.448	--	--

The effect of the different treatment groups does not depend upon the value of covariate Day, averaging over the values of the remaining covariates. There is not a significant interaction between the factor Sample and the covariate Day (P = 0.561).
There are no significant interactions between the factor and the covariates. The equal slopes assumption passes and the equal slopes model is analyzed below.
Analysis of Equal Slopes Model:
R = 0.788 Rsqr = 0.622 Adj Rsqr = 0.537
Analysis of Variance for the Equal Slopes Model:

Source of Variation	DF	SS	MS	F	P
Sample	1	128.524	128.524	13.590	0.005
Day	1	11.283	11.283	1.193	0.303
Residual	9	85.117	9.457	--	--
Total	11	224.925	20.448	--	--

The differences in the adjusted means among the treatment groups are greater than would be expected by chance; there is a statistically significant difference (P = 0.005). To isolate which group(s) differ most from the others use a multiple comparison procedure. The adjusted means and their statistics are given in the table below.
The coefficient of covariate Day in the equal slopes regression model is not significantly different from zero (P = 0.303). There is no evidence that the covariate affects the values of dependent variable; you may want to consider removing it from the regression model.
No covariate significantly contributes to the values of the dependent variable. You may want to consider a single factor ANOVA design for your study.
Adjusted Means of the Groups:

Group Name	Adjusted Mean	Std. Error	95%Conf-L	95%Conf-U
KD	1.876	1.255	-0.964	4.716
N	-4.669	1.255	-7.509	-1.829

The adjusted means are the predicted values of the dependent variable Col 4 for each group where each covariate variable is evaluated at the average of its data values.
Multiple Comparisons versus Control Group (Holm-Sidak method):
Comparisons for factor: **Sample**

Comparison	Diff of Means	t	P	P<0.050
N vs. KD	6.545	3.686	0.005	Yes

Regression Equations for the Equal Slopes Model:
There is a significant difference in the intercepts of the dependent variable for these equations since there is a significant difference in the adjusted means of the factor groups (P = 0.005).
Group: KD
Col 4 = 4.139 - (0.646 * Day)
Group: N
Col 4 = -2.407 - (0.646 * Day)

One Way Analysis of Covariance Dependent Variable: dCt_KD

Data source: Data 1

**Group Name**	**N**	**Missing**	**Mean**	**Std Dev**	**SEM**
KD	5	0	3.333	1.255	0.561
N	6	0	-4.669	2.297	0.938
Total	11	0	-1.032	4.554	1.373

Normality Test (Shapiro-Wilk): Passed (P = 0.344)
Equal Variance Test (Levene): Passed (P = 0.173)
Equal Slopes Test: Passed (P = 0.811)
Analysis of Variance for the Interaction Model:

**Source of Variation**	**DF**	**SS**	**MS**	**F**	**P**
Sample_KD	1	23.516	23.516	5.203	0.057
Day_KD	1	0.666	0.666	0.147	0.713
Sample_KD x Day_KD	1	0.280	0.280	0.0619	0.811
Residual	7	31.640	4.520	--	--
Total	10	207.352	20.735	--	--

The effect of the different treatment groups does not depend upon the value of covariate Day_KD, averaging over the values of the remaining covariates. There is not a significant interaction between the factor Sample_KD and the covariate Day_KD (P = 0.811).
There are no significant interactions between the factor and the covariates. The equal slopes assumption passes and the equal slopes model is analyzed below.
Analysis of Equal Slopes Model:
R = 0.920 Rsqr = 0.846 Adj Rsqr = 0.808
Analysis of Variance for the Equal Slopes Model:

**Source of Variation**	**DF**	**SS**	**MS**	**F**	**P**
Sample_KD	1	170.604	170.604	42.758	<0.001
Day_KD	1	0.775	0.775	0.194	0.671
Residual	8	31.920	3.990	--	--
Total	10	207.352	20.735	--	--

The differences in the adjusted means among the treatment groups are greater than would be expected by chance; there is a statistically significant difference (P = <0.001). To isolate which group(s) differ most from the others use a multiple comparison procedure. The adjusted means and their statistics are given in the table below.
The coefficient of covariate Day_KD in the equal slopes regression model is not significantly different from zero (P = 0.671). There is no evidence that the covariate affects the values of dependent variable; you may want to consider removing it from the regression model.
No covariate significantly contributes to the values of the dependent variable. You may want to consider a single factor ANOVA design for your study.
Adjusted Means of the Groups:

**Group Name**	**Adjusted Mean**	**Std. Error**	**95%Conf-L**	**95%Conf-U**
KD	3.304	0.896	1.239	5.370
N	-4.645	0.817	-6.530	-2.760

The adjusted means are the predicted values of the dependent variable Col 17 for each group where each covariate variable is evaluated at the average of its data values.
Multiple Comparisons versus Control Group (Holm-Sidak method):
Comparisons for factor: **Sample_KD**

**Comparison**	**Diff of Means**	**t**	**P**	**P<0.050**
N vs. KD	7.949	6.539	<0.001	Yes

Regression Equations for the Equal Slopes Model:
There is a significant difference in the intercepts of the dependent variable for these equations since there is a significant difference in the adjusted means of the factor groups (P = <0.001).
Group: KD
Col 17 = 3.905 - (0.179 * Day_KD)
Group: N
Col 17 = -4.044 - (0.179 * Day_KD)

One Way Analysis of Covariance – Dependent Variable: C#/C#
Data source: Data 1

**Group Name**	**N**	**Missing**	**Mean**	**Std Dev**	**SEM**
KD	6	0	5.221	12.059	4.923
N	6	0	33.693	32.575	13.298
Total	12	0	19.457	27.740	8.008

Normality Test (Shapiro-Wilk): Passed (P = 0.636)
Equal Variance Test (Levene): Failed (P < 0.050)
Equal Slopes Test: Passed (P =0.839)

Analysis of Variance for the Interaction Model:

**Source of Variation**	**DF**	**SS**	**MS**	**F**	**P**
Sample	1	207.022	207.022	0.302	0.598
Day	1	512.119	512.119	0.746	0.413
Sample x Day	1	30.046	30.046	0.0438	0.839
Residual	8	5490.418	686.302	--	--
Total	11	8464.635	769.512	--	--

The effect of the different treatment groups does not depend upon the value of covariate Day, averaging over the values of the remaining covariates. There is not a significant interaction between the factor Sample and the covariate Day (P = 0.839).
There are no significant interactions between the factor and the covariates. The equal slopes assumption passes and the equal slopes model is analyzed below.

Analysis of Equal Slopes Model:
R = 0.590 Rsqr = 0.348 Adj Rsqr = 0.203
Analysis of Variance for the Equal Slopes Model:

**Source of Variation**	**DF**	**SS**	**MS**	**F**	**P**
Sample	1	2432.052	2432.052	3.965	0.078
Day	1	512.119	512.119	0.835	0.385
Residual	9	5520.464	613.385	--	--
Total	11	8464.635	769.512	--	--

The differences among the adjusted means of the treatment groups are not great enough to exclude the possibility that the differences are only due to random sample variability. There is not a significant difference between the adjusted means (P = 0.078).
The coefficient of covariate Day in the equal slopes regression model is not significantly different from zero (P = 0.385). There is no evidence that the covariate affects the values of dependent variable; you may want to consider removing it from the regression model.
No covariate significantly contributes to the values of the dependent variable. You may want to consider a single factor ANOVA design for your study.

Adjusted Means of the Groups:

**Group Name**	**Adjusted Mean**	**Std. Error**	**95%Conf-L**	**95%Conf-U**
KD	5.221	10.111	-17.652	28.093
N	33.693	10.111	10.821	56.566

The adjusted means are the predicted values of the dependent variable C#/C# for each group where each covariate variable is evaluated at the average of its data values.
Regression Equations for the Equal Slopes Model:
There is no significant difference in the intercepts of the dependent variable for these equations since there is no significant difference in the adjusted means of the factor groups (P = 0.078).
Group: KD
C#/C# = -10.022 + (4.355 * Day)
Group: N
C#/C# = 18.450 + (4.355 * Day)

One Way Analysis of Covariance – Dependent Variable: log(C#/C#)
Data source: Data 1

Group Name	N	Missing	Mean	Std Dev	SEM
KD	6	0	-0.583	2.042	0.833
N	6	0	2.980	1.241	0.507
Total	12	0	1.198	2.461	0.710

Normality Test (Shapiro-Wilk): Passed (P = 0.414)
Equal Variance Test (Levene): Passed (P = 0.687)
Equal Slopes Test: Passed (P = 0.560)
Analysis of Variance for the Interaction Model:

Source of Variation	DF	SS	MS	F	P
Sample	1	11.546	11.546	3.861	0.085
Day	1	3.504	3.504	1.172	0.311
Sample x Day	1	1.107	1.107	0.370	0.560
Residual	8	23.925	2.991	--	--
Total	11	66.620	6.056	--	--

The effect of the different treatment groups does not depend upon the value of covariate Day, averaging over the values of the remaining covariates. There is not a significant interaction between the factor Sample and the covariate Day (P = 0.560).
There are no significant interactions between the factor and the covariates. The equal slopes assumption passes and the equal slopes model is analyzed below.
Analysis of Equal Slopes Model:
R = 0.790   Rsqr = 0.624   Adj Rsqr = 0.541
Analysis of Variance for the Equal Slopes Model:

Source of Variation	DF	SS	MS	F	P
Sample	1	38.084	38.084	13.693	0.005
Day	1	3.504	3.504	1.260	0.291
Residual	9	25.032	2.781	--	--
Total	11	66.620	6.056	--	--

The differences in the adjusted means among the treatment groups are greater than would be expected by chance; there is a statistically significant difference (P = 0.005). To isolate which group(s) differ most from the others use a multiple comparison procedure. The adjusted means and their statistics are given in the table below.
The coefficient of covariate Day in the equal slopes regression model is not significantly different from zero (P = 0.291). There is no evidence that the covariate affects the values of dependent variable; you may want to consider removing it from the regression model.
No covariate significantly contributes to the values of the dependent variable. You may want to consider a single factor ANOVA design for your study.
Adjusted Means of the Groups:

Group Name	Adjusted Mean	Std. Error	95%Conf-L	95%Conf-U
KD	-0.583	0.681	-2.124	0.957
N	2.980	0.681	1.439	4.520

The adjusted means are the predicted values of the dependent variable log(C#/C#) for each group where each covariate variable is evaluated at the average of its data values.
All Pairwise Multiple Comparison Procedures (Holm-Sidak method):
Comparisons for factor: **Sample**

Comparison	Diff of Means	t	P	P<0.050
N vs. KD	3.563	3.700	0.005	Yes

Regression Equations for the Equal Slopes Model:
There is a significant difference in the intercepts of the dependent variable for these equations since there is a significant difference in the adjusted means of the factor groups (P = 0.005).
Group: KD
log(C#/C#) = -1.844 + (0.360 * Day)
Group: N
log(C#/C#) = 1.719 + (0.360 * Day)

One Way Analysis of Covariance – Dependent Variable: log(2^dCt)_KD
Data source: Data 1

Group Name	N	Missing	Mean	Std Dev	SEM
KD	5	0	-1.772	0.667	0.298
N	6	0	2.482	1.221	0.499
Total	11	0	0.548	2.420	0.730

Normality Test (Shapiro-Wilk): Passed (P = 0.344)
Equal Variance Test (Levene): Passed (P = 0.173)
Equal Slopes Test: Passed (P = 0.811)
Analysis of Variance for the Interaction Model:

Source of Variation	DF	SS	MS	F	P
Sample_KD	1	6.645	6.645	5.203	0.057
Day_KD	1	0.188	0.188	0.147	0.712
Sample_KD x Day_KD	1	0.0790	0.0790	0.0618	0.811
Residual	7	8.940	1.277	--	--
Total	10	58.585	5.859	--	--

The effect of the different treatment groups does not depend upon the value of covariate Day_KD, averaging over the values of the remaining covariates. There is not a significant interaction between the factor Sample_KD and the covariate Day_KD (P = 0.811).
There are no significant interactions between the factor and the covariates. The equal slopes assumption passes and the equal slopes model is analyzed below.
Analysis of Equal Slopes Model:
R = 0.920 Rsqr = 0.846 Adj Rsqr = 0.808
Analysis of Variance for the Equal Slopes Model:

Source of Variation	DF	SS	MS	F	P
Sample_KD	1	48.202	48.202	42.758	<0.001
Day_KD	1	0.219	0.219	0.194	0.671
Residual	8	9.019	1.127	--	--
Total	10	58.585	5.859	--	--

The differences in the adjusted means among the treatment groups are greater than would be expected by chance; there is a statistically significant difference (P = <0.001). To isolate which group(s) differ most from the others use a multiple comparison procedure. The adjusted means and their statistics are given in the table below.
The coefficient of covariate Day_KD in the equal slopes regression model is not significantly different from zero (P = 0.671). There is no evidence that the covariate affects the values of dependent variable; you may want to consider removing it from the regression model.
No covariate significantly contributes to the values of the dependent variable. You may want to consider a single factor ANOVA design for your study.
Adjusted Means of the Groups:

Group Name	Adjusted Mean	Std. Error	95%Conf-L	95%Conf-U
KD	-1.756	0.476	-2.854	-0.658
N	2.469	0.434	1.467	3.471

The adjusted means are the predicted values of the dependent variable log(2^dCt)_KD for each group where each covariate variable is evaluated at the average of its data values.
All Pairwise Multiple Comparison Procedures (Holm-Sidak method):
Comparisons for factor: **Sample_KD**

Comparison	Diff of Means	t	P	P<0.050
N vs. KD	4.225	6.539	<0.001	Yes

Regression Equations for the Equal Slopes Model:
There is a significant difference in the intercepts of the dependent variable for these equations since there is a significant difference in the adjusted means of the factor groups (P = <0.001).
Group: KD
log(2^dCt)_KD = -2.076 + (0.0949 * Day_KD)
Group: N
log(2^dCt)_KD = 2.150 + (0.0949 * Day_KD)

One Way Analysis of Covariance – Dependent Variable: log(C#/C#)_KD
Data source: Data 1

**Group Name**	**N**	**Missing**	**Mean**	**Std Dev**	**SEM**
KD	5	0	-1.379	0.678	0.303
N	6	0	2.980	1.241	0.507
Total	11	0	0.998	2.477	0.747

Normality Test (Shapiro-Wilk): Passed (P = 0.418)
Equal Variance Test (Levene): Passed (P = 0.176)
Equal Slopes Test: Passed (P = 0.809)
Analysis of Variance for the Interaction Model:

**Source of Variation**	**DF**	**SS**	**MS**	**F**	**P**
Sample_KD	1	6.968	6.968	5.312	0.055
Day_KD	1	0.235	0.235	0.179	0.685
Sample_KD x Day_KD	1	0.0830	0.0830	0.0632	0.809
Residual	7	9.183	1.312	--	--
Total	10	61.352	6.135	--	--

The effect of the different treatment groups does not depend upon the value of covariate Day_KD, averaging over the values of the remaining covariates. There is not a significant interaction between the factor Sample_KD and the covariate Day_KD (P = 0.809).
There are no significant interactions between the factor and the covariates. The equal slopes assumption passes and the equal slopes model is analyzed below.
Analysis of Equal Slopes Model:
R = 0.921 Rsqr = 0.849 Adj Rsqr = 0.811
Analysis of Variance for the Equal Slopes Model:

**Source of Variation**	**DF**	**SS**	**MS**	**F**	**P**
Sample_KD	1	50.555	50.555	43.649	<0.001
Day_KD	1	0.271	0.271	0.234	0.642
Residual	8	9.266	1.158	--	--
Total	10	61.352	6.135	--	--

The differences in the adjusted means among the treatment groups are greater than would be expected by chance; there is a statistically significant difference (P = <0.001). To isolate which group(s) differ most from the others use a multiple comparison procedure. The adjusted means and their statistics are given in the table below.
The coefficient of covariate Day_KD in the equal slopes regression model is not significantly different from zero (P = 0.642). There is no evidence that the covariate affects the values of dependent variable; you may want to consider removing it from the regression model.
No covariate significantly contributes to the values of the dependent variable. You may want to consider a single factor ANOVA design for your study.
Adjusted Means of the Groups:

**Group Name**	**Adjusted Mean**	**Std. Error**	**95%Conf-L**	**95%Conf-U**
KD	-1.362	0.483	-2.475	-0.249
N	2.965	0.440	1.950	3.981

The adjusted means are the predicted values of the dependent variable log(C#/C#)_KD for each group where each covariate variable is evaluated at the average of its data values.
All Pairwise Multiple Comparison Procedures (Holm-Sidak method):
Comparisons for factor: **Sample_KD**

**Comparison**	**Diff of Means**	**t**	**P**	**P<0.050**
N vs. KD	4.327	6.607	<0.001	Yes

Regression Equations for the Equal Slopes Model:
There is a significant difference in the intercepts of the dependent variable for these equations since there is a significant difference in the adjusted means of the factor groups (P = <0.001).
Group: KD
log(C#/C#)_KD = -1.717 + (0.106 * Day_KD)
Group: N
log(C#/C#)_KD = 2.610 + (0.106 * Day_KD)

Analysis of qPCR data in R

Summary: There have been works describing the use of linear (1) and generalized linear (2) models with qPCR data for relative expression quantification, a nd w hen q uantifying m icrobiomes (3, 4). H ere, we compared typical methods of analysis with the use of a negative binomial model (i.e. transformed, normally distributed data vs. raw, non-normal data). Encouragingly, $logE^{-\Delta Ct}$, $log(C\#_{TetR}/C\#_{16S})$, and lm methods produced similar mean relative expression results, showing consistency when implementing similar methods and software (Table 1 and S11). The prediction of mean $C\#_{TetR}/C\#_{16S}$ was found to be more representative of arithmetic means using glm.nb method (Table 1 and S11), while both prediction of means and relative expression were more sensitive to the outlier than other methods. The difference in arithmetic and predicted means is likely to be a result of log-transformation (5) (Supplementary Table S8), a potential benefit of using negative binomial models. However, further validation would be useful, and ultimately the model used depends on what needs to be described (6). For example, if qPCR analysis included a comparison of sgRNA guide effectiveness (i.e. more than one guide, as for 2,3-BDO formation, and acetone to isopropanol production), more accurate mean relative expression (smaller CIs) could be useful for quantifying KD effectiveness. Yet, removal of outliers appears to affect CIs more than selection of method (Table 1 and S11). Further improvements to mean relative expression accuracy are obtainable by using R packages for mixed-effect models (7) or variance-covariance matrix estimators that have adjustments for heteroskedasticity and small-sample performance (8) (Supplementary Table S11). In general, although there may be some benefits to using negative binomial models, thoroughness is recommended as qPCR, and its analysis, are still complex (9).

References

1. Steibel,J.P., Poletto,R., Coussens,P.M. and Rosa,G.J.M. (2009) A powerful and flexible linear mixed model framework for the analysis of relative quantification RT-PCR data. Genomics, 94, 146–152.
2. Matz,M. V., Wright,R.M. and Scott,J.G. (2013) No Control Genes Required: Bayesian Analysis of qRT-PCR Data. PLoS One, 8, e71448.
3. Jian,C., Luukkonen,P., Yki-Järvinen,H., Salonen,A. and Korpela,K. (2020) Quantitative PCR provides a simple and accessible method for quantitative microbiota profiling. PLoS One, 15, e0227285.
4. McMurdie,P.J. and Holmes,S. (2014) Waste Not, Want Not: Why Rarefying Microbiome Data Is Inadmissible. PLoS Comput. Biol., 10, e1003531.
5. Russell,C.J. and Dean,M.A. (2000) To Log or Not to Log: Bootstrap as an Alternative to the Parametric Estimation of Moderation Effects in the Presence of Skewed Dependent Variables. Organ. Res. Methods, 3, 166–185.
6. Manning,W.G. and Mullahy,J. (2001) Estimating log models: To transform or not to transform? J. Health Econ., 20, 461–494.
7. Bates,D., Mächler,M., Bolker,B.M. and Walker,S.C. (2015) Fitting linear mixed-effects models using lme4. J. Stat. Softw., 67, 1–48.
8. Pustejovsky,J.E. and Tipton,E. (2018) Small-Sample Methods for Cluster-Robust Variance Estimation and Hypothesis Testing in Fixed Effects Models. J. Bus. Econ. Stat., 36, 672–683.
9. Taylor,S.C., Nadeau,K., Abbasi,M., Lachance,C., Nguyen,M. and Fenrich,J. (2019) The Ultimate qPCR Experiment: Producing Publication Quality, Reproducible Data the First Time. Trends Biotechnol., 37, 761–774.

Loading package libraries and data

```
library(tidyverse)
library(ggpubr)
library(pastecs)
library(clubSandwich)
library(msm)
library(car)
library(lme4)
library(performance)
library(DHARMa)
library(MASS)
library(merDeriv)
library(stargazer)
```

File path not shown for privacy, data frames printed below and displayed in Supplementary Tables S9 & 10

Example code:

```
copydata <- read.csv("C:/...path.../filename.csv")
copyratioT <- read.csv("C:/...path.../filename1.csv")
```

Manipulating data for various uses

```
copydata
```

```
##      Gene Day Sample Bioreplicate    Copy log2Copy   CorCopy
## 1   tetR   2     KD          BR1  109480 16.74031   2383646
## 2   tetR   2     KD          BR2  636700 19.28025   2232287
## 3   tetR   2     KD          BR3  390825 18.57616    757485
## 4   tetR   2      N          BR1   40775 15.31540  29056134
## 5   tetR   2      N          BR2  220150 17.74813  94665741
## 6   tetR   2      N          BR3  102223 16.64136 374009955
## 7   tetR   5     KD          BR1  142775 17.12338 186728010
## 8   tetR   5     KD          BR2  138775 17.08239   3611457
## 9   tetR   5     KD          BR3   94330 16.52543    886739
## 10  tetR   5      N          BR1  192200 17.55225 216531668
## 11  tetR   5      N          BR2  101705 16.63403 523295438
## 12  tetR   5      N          BR3   75198 16.19841  33526666
## 13   16S   2     KD          BR1  301306 18.20087        NA
## 14   16S   2     KD          BR2 1871111 20.83546        NA
## 15   16S   2     KD          BR3 3384722 21.69061        NA
## 16   16S   2      N          BR1    9206 13.16836        NA
## 17   16S   2      N          BR2   15256 13.89709        NA
## 18   16S   2      N          BR3    1793 10.80816        NA
## 19   16S   5     KD          BR1    5016 12.29232        NA
## 20   16S   5     KD          BR2  252083 17.94354        NA
## 21   16S   5     KD          BR3  697861 19.41258        NA
## 22   16S   5      N          BR1    5823 12.50755        NA
## 23   16S   5      N          BR2    1275 10.31628        NA
## 24   16S   5      N          BR3   14714 13.84490        NA
```

```
copydata_wide  <-  spread(copydata[,-c(6,7)],Gene,Copy)
copydata$Comparison <- paste(copydata$Sample,copydata$Day)
copydata$logCopy <- log(copydata$Copy)
copydata_wide$Comparison <- paste(copydata_wide$Sample,copydata_wide$Day)
copydata_wide$CorCopy  <-  copydata$CorCopy[1:12]

copyratioT
```

```
##      Day Sample Bioreplicate          T1          T2.1         T2.2
## 1      2     KD          BR1   0.2993426     0.4682785    0.3835294
## 2      2     KD          BR2   0.3037336     0.3794904    0.3528992
## 3      2     KD          BR3   0.1035688     0.1297302    0.1178615
## 4      2      N          BR1   3.6882840     5.3685786    4.8957176
## 5      2      N          BR2  10.8281250    19.6221837   17.7417504
## 6      2      N          BR3  48.9904031    84.0039578   48.2930464
## 7      5     KD          BR1  18.9968467    39.0175953   42.3238145
## 8      5     KD          BR2   0.4995475     0.6226254    0.5602874
## 9      5     KD          BR3   0.1261661     0.1539073    0.1309235
## 10     5      N          BR1  23.9114391    46.8385027   43.0407883
## 11     5      N          BR2  51.2218868   131.3987022  109.1523895
## 12     5      N          BR3   3.7557331     7.0865009    6.4058005
```

```
copynumRT_t  <-  copyratioT[,-c(1,2,3)]
copynumRT_t <- data.frame(t(copynumRT_t))
copyRTstat  <-  stat.desc(rbind(copynumRT_t[1,],slice(stat.desc(copynumRT_t[2:3,]),9)))
```

```
pointdata <- data.frame(
  t(slice(copyRTstat,9)),
  t(slice(copyRTstat,13)))
pointdata <- cbind(copyratioT,pointdata)
pointdata$logmean <- log(pointdata$mean)

#Removing potential outlier (KD day 5 BR1)
pointdata_KD  <- pointdata[-7,]
copydata_KD  <- copydata[-c(7,19),]
copydata_wide_KD  <- copydata_wide[-7,]

pointdata$Comparison <- paste(pointdata$Sample,"Day",pointdata$Day)
pointdata$Comparison2 <- paste(pointdata$Sample,pointdata$Bioreplicate)
copydata_wide$Comparison2 <- paste(copydata_wide$Sample, copydata_wide$Bioreplicate)
pointdata_KD  <-  pointdata[-7,]
copydata_KD  <- copydata[-c(7,19),]
copydata_wide_KD  <- copydata_wide[-7,]

pointav  <-  cbind(pointdata[1:2],pointdata[11],pointdata[10],
                   Mean = c(
  rep(mean(subset(pointdata, Comparison == "KD Day 2", select = "mean")[1:3,]),3),
  rep(mean(subset(pointdata, Comparison == "N Day 2", select = "mean")[1:3,]),3),
  rep(mean(subset(pointdata, Comparison == "KD Day 5", select = "mean")[1:3,]),3),
  rep(mean(subset(pointdata, Comparison == "N Day 5", select = "mean")[1:3,]),3)),
                   MeanSample = c(rep(mean(subset(pointdata, Sample == "KD",
                                              select = "mean")[1:6,]),3),
                                  rep(mean(subset(pointdata, Sample == "N",
                                              select = "mean")[1:6,]),3),
                                  rep(mean(subset(pointdata, Sample == "KD",
                                              select = "mean")[1:6,]),3),
                                  rep(mean(subset(pointdata, Sample == "N",
                                              select = "mean")[1:6,]),3)),
                   logMean = c(
  rep(mean(subset(pointdata, Comparison == "KD Day 2", select = "logmean")[1:3,]),3),
  rep(mean(subset(pointdata, Comparison == "N Day 2", select = "logmean")[1:3,]),3),
  rep(mean(subset(pointdata, Comparison == "KD Day 5", select = "logmean")[1:3,]),3),
  rep(mean(subset(pointdata, Comparison == "N Day 5", select = "logmean")[1:3,]),3)),
                   logMeanSample = c(rep(mean(subset(pointdata, Sample == "KD",
                                                select = "logmean")[1:6,]),3),
                                  rep(mean(subset(pointdata, Sample == "N",
                                              select = "logmean")[1:6,]),3),
                                  rep(mean(subset(pointdata, Sample == "KD",
                                              select = "logmean")[1:6,]),3),
                                  rep(mean(subset(pointdata, Sample == "N",
                                              select = "logmean")[1:6,]),3))
)
```

Graphing data

```
#Generating Figure 4

dodge <- position_dodge(.6)

pointdata %>%
  ggplot(aes(x=Comparison2,y=mean,group=factor(Day))) +
  geom_line(data = pointav, aes(y = MeanSample, group=factor(Comparison)), size = 1) +
  geom_line(data = pointav, aes(y = Mean, group=factor(Comparison)), size = 1.5) +
  geom_line(data = pointav, aes(y = Mean, group=factor(Comparison), colour = Comparison),
            size = 1) +
  geom_errorbar(aes(ymin = mean-std.dev, ymax=mean+std.dev), width=.2,
                 position=dodge) +
  geom_point(
    size = 3,
    position=dodge,
    aes(colour = Comparison)
    ) +
  scale_colour_brewer(palette = "Paired") +
  geom_point(
    shape = 1,
    size = 3,
    position=dodge,
    colour = "black"
    ) +
  scale_y_log10() +
  theme_classic2() +
  theme(legend.position = "right",
        plot.title = element_text(size = 11)) +
  xlab("")
```

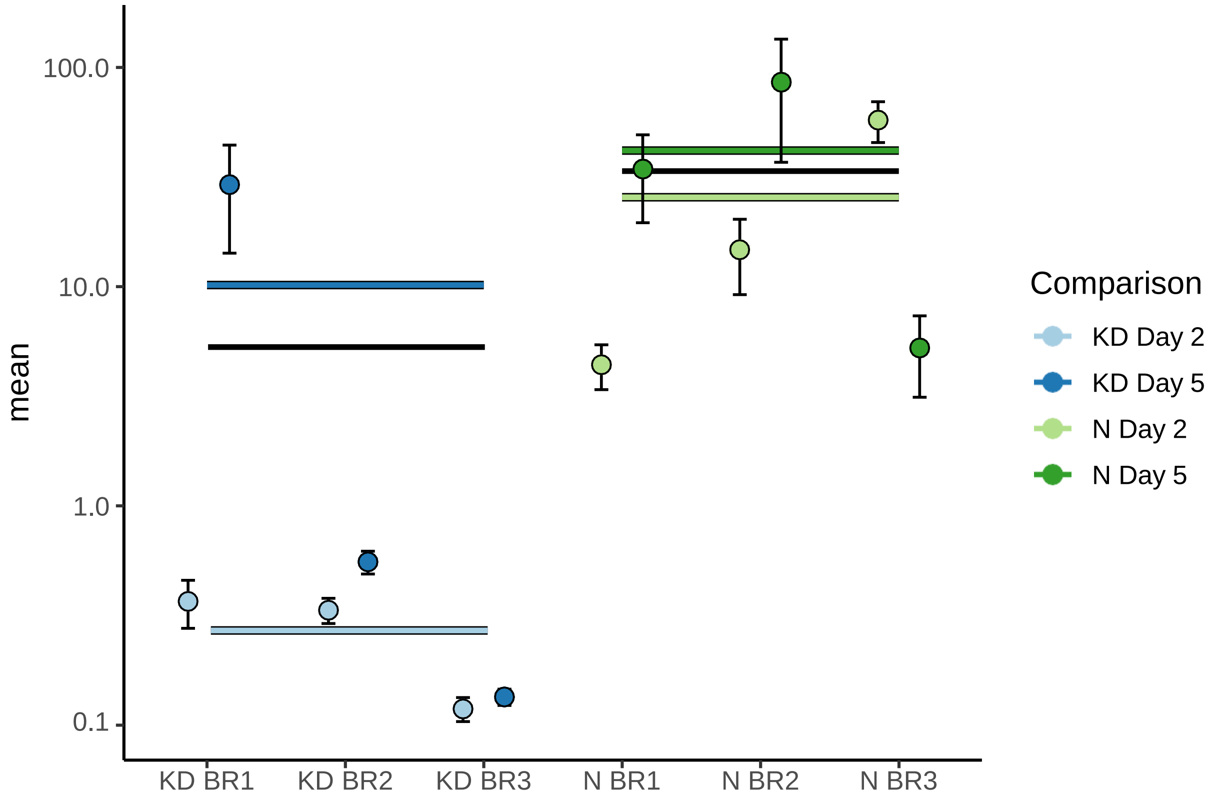
100.0
10.0
1.0
0.1
mean
KD BR1
KD BR2
KD BR3
N BR1
N BR2
N BR3
Comparison
KD Day 2
KD Day 5
N Day 2
N Day 5

Assessing normality

```r
#Plotting histogram to visualise distribution of data.
#histogram of 1Copy1
ggplot(copydata, aes(Copy, fill = Sample)) + geom_histogram() + facet_grid(Sample ~
    ., margins = TRUE, scales = "free") + ggtitle('Copy number data')
```

```
## 'stat_bin()' using 'bins = 30'. Pick better value with 'binwidth'.
```

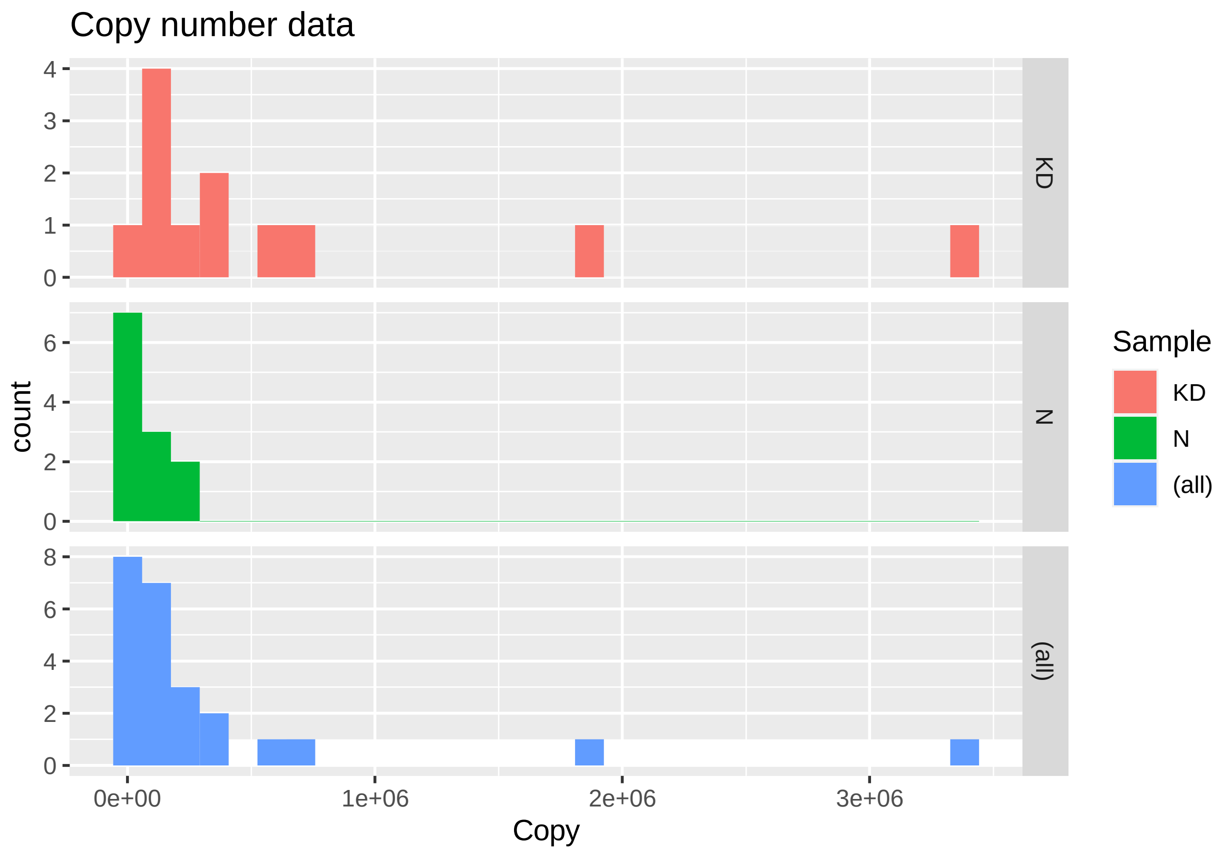


```r
#histogram of 1Ratio1
ggplot(pointdata, aes(mean, fill = Sample)) + geom_histogram() + facet_grid(Sample ~
    ., margins = TRUE, scales = "free") + ggtitle('Copy number ratio data')
```

```
## 'stat_bin()' using 'bins = 30'. Pick better value with 'binwidth'.
```

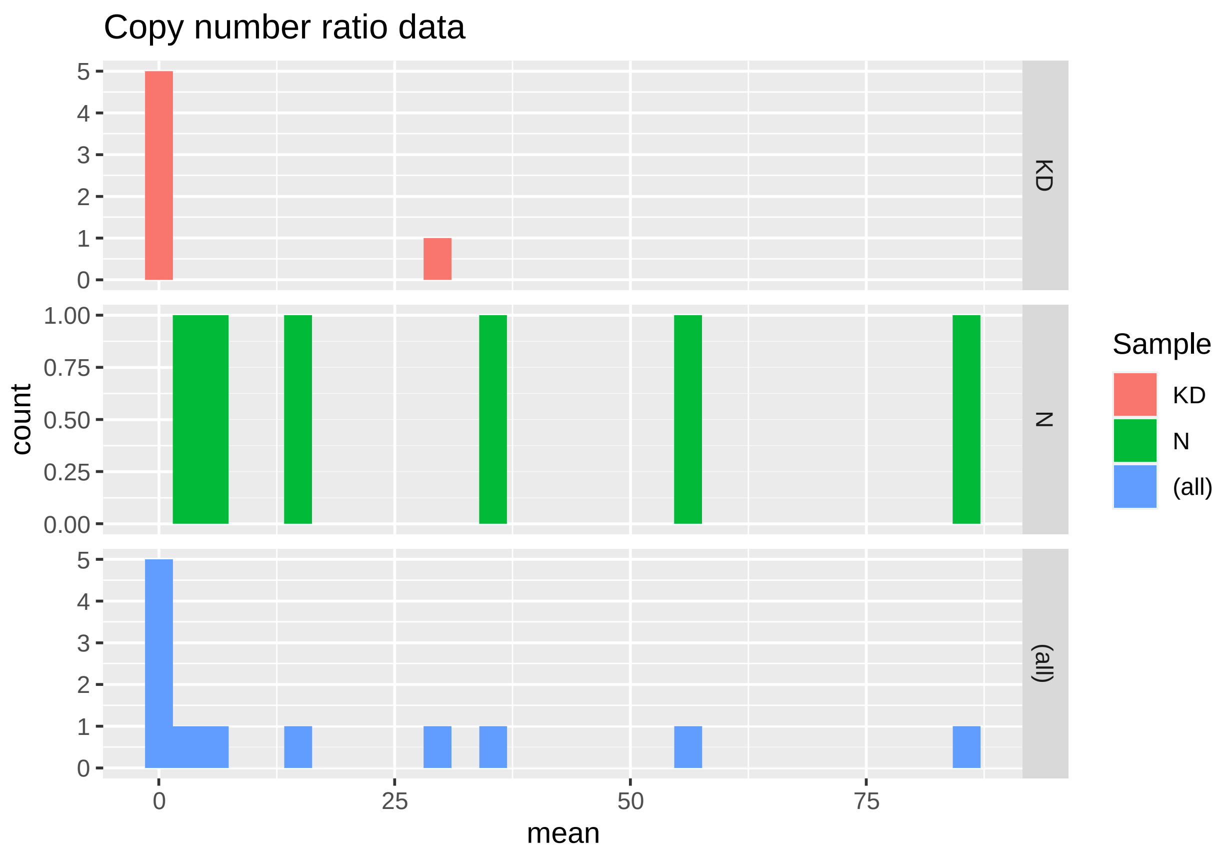


```r
#Quantile-Quantile (Q-Q) plots correlate sample to normal distribution
ggqqplot(copydata$Copy) +
  ggtitle("'Copy#'  Q-Q plot")
```

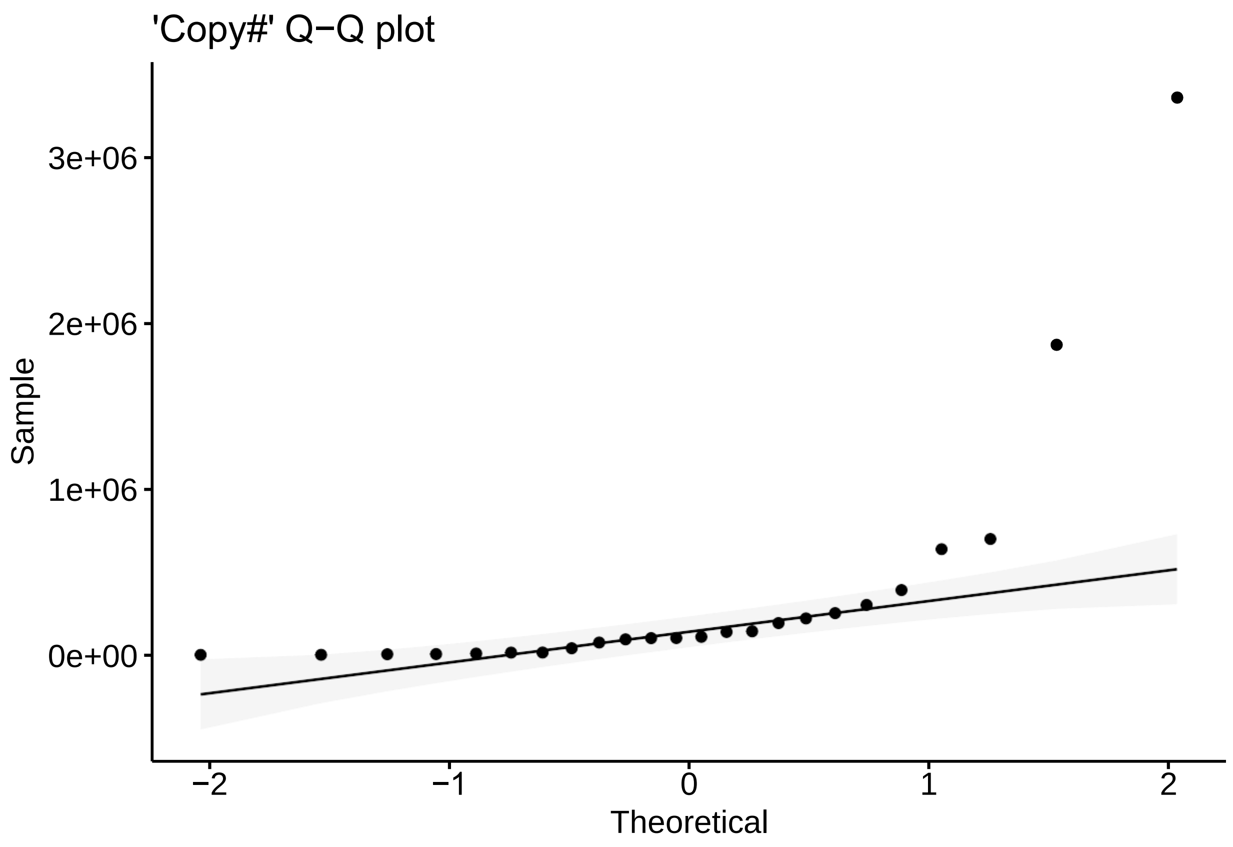


```
ggqqplot(copydata$logCopy) +
  ggtitle("log(Copy#) Q-Q plot")
```

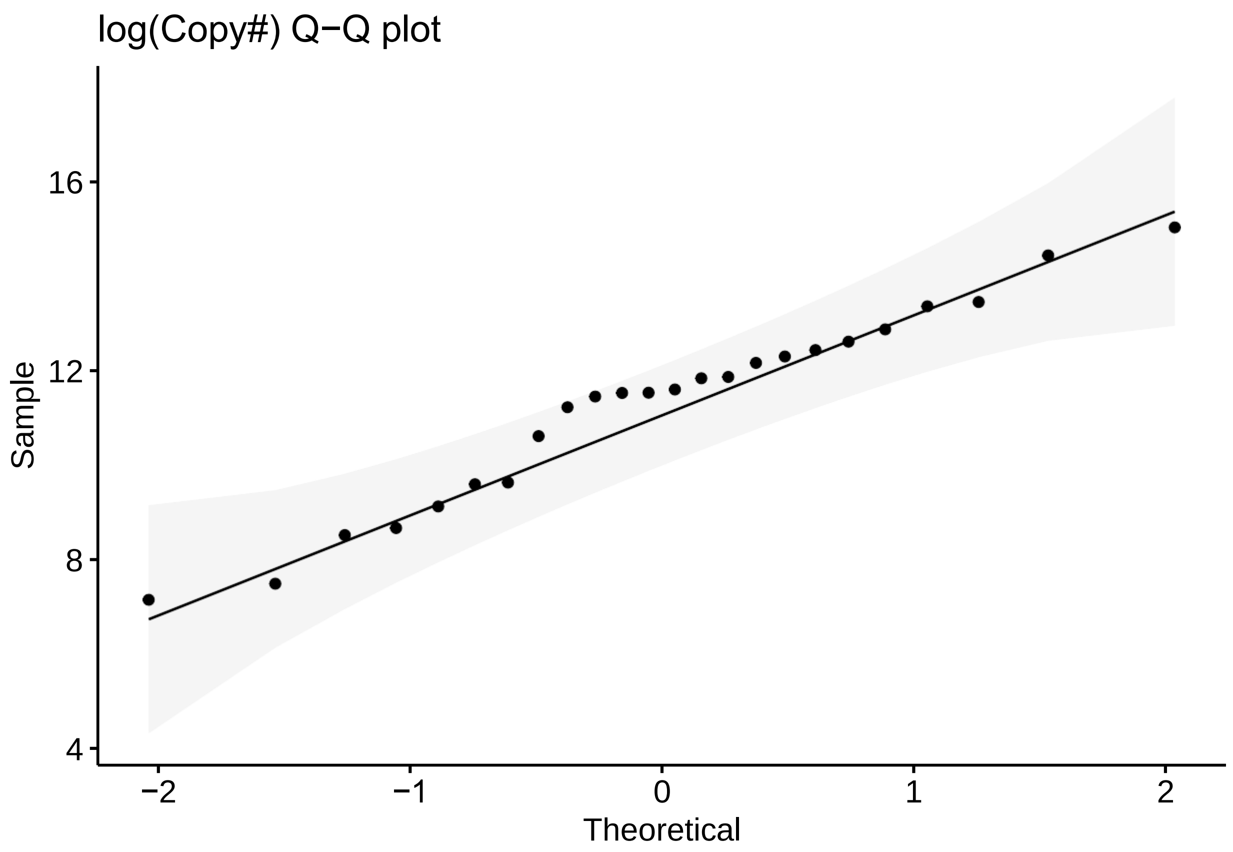


```
ggqqplot(pointdata$mean) +
  ggtitle("'Copy# Ratio' Q-Q plot")
```

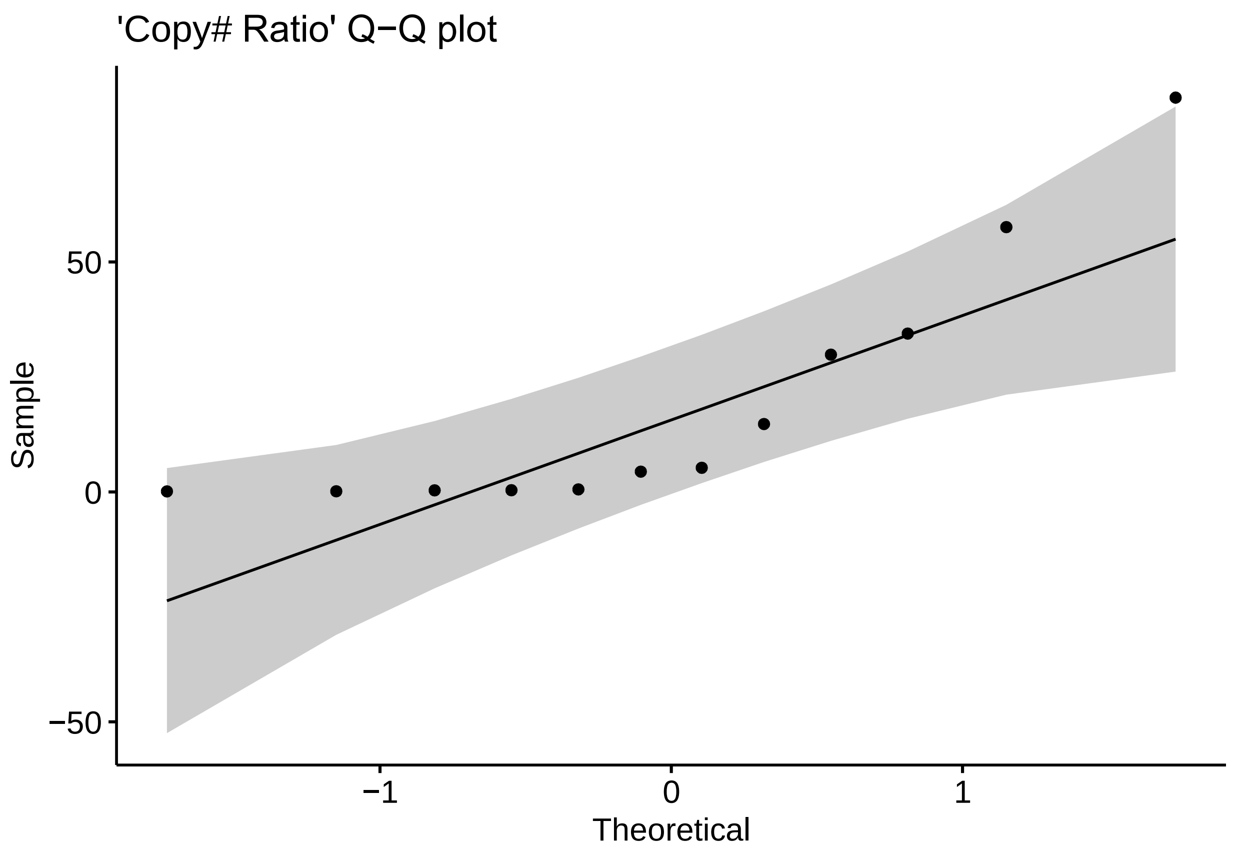


```
ggqqplot(pointdata$logmean) +
  ggtitle("log(Copy# Ratio) Q-Q plot")
```

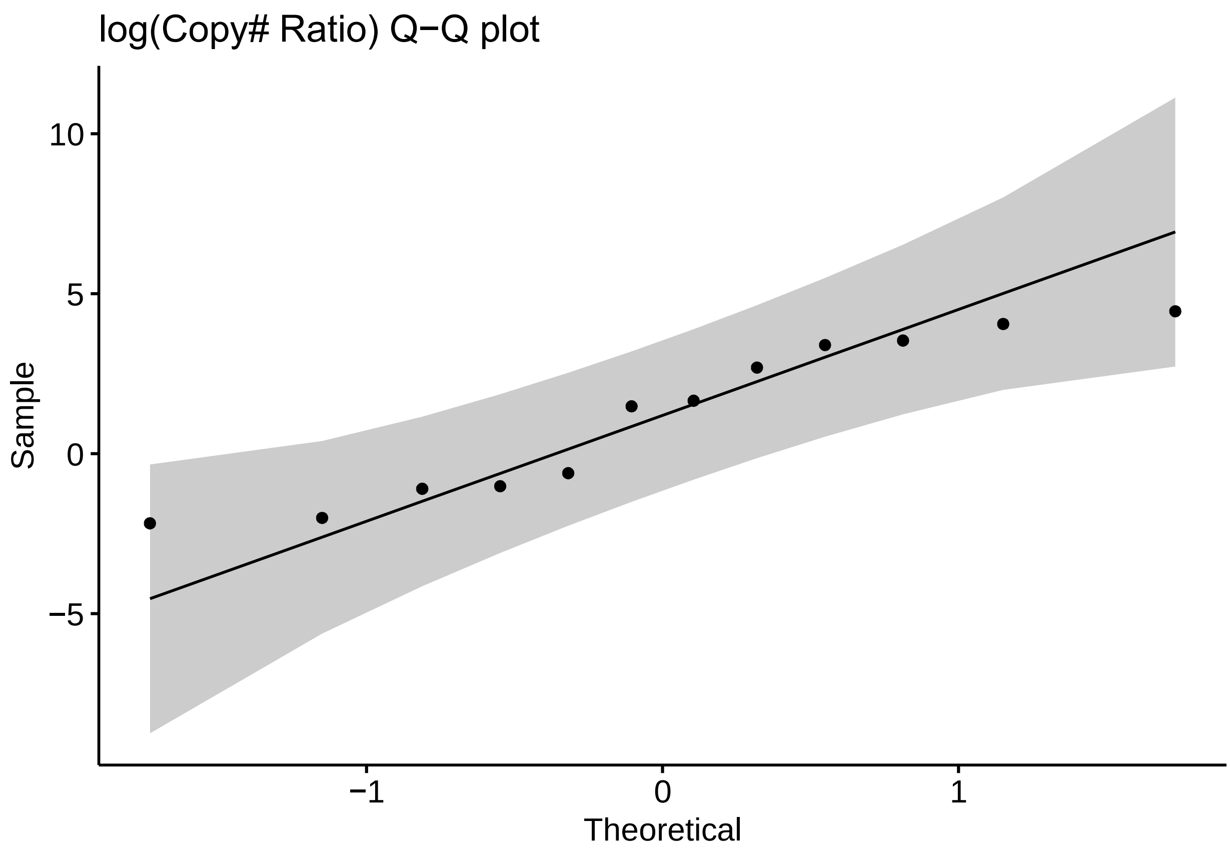


```
#1Copy1  and 1Ratio1 data appear to be non-normal, so the normality is tested
shapiro.test(copydata$Copy)
```

```
##
##  Shapiro-Wilk normality test
##
## data:  copydata$Copy
## W = 0.5095, p-value = 6.982e-08
```

```
shapiro.test(copydata$logCopy)
```

```
##
##  Shapiro-Wilk normality test
##
## data:  copydata$logCopy
## W = 0.96429, p-value = 0.5305
```

```
shapiro.test(pointdata$mean)
```

```
##
##  Shapiro-Wilk normality test
##
## data:  pointdata$mean
## W = 0.75604, p-value = 0.003092
```

```
shapiro.test(pointdata$logmean)
```

```
##
##  Shapiro-Wilk normality test
```

```
##
## data:   pointdata$logmean
## W = 0.89924, p-value = 0.1551
```

```
#All normality tests evidence 1Copy#1  and 1Ratio1  data are not normally
#distributed, while log transformed data is.
```

Generalised linear model (Poisson)

```r
#Poisson distributed models have greater constraints (as mean = var),
#therefore this was modeled before negative binomial.
#Generate Poisson model - here the ratio is generated using an offset,
#as Poisson models require non-negative integers
m1 <- glm(tetR ~ Sample + Day  + offset(log(copydata_wide$"16S"))
              , data = copydata_wide
              , family = "poisson"
              )
summary(m1)
```

```
##
## Call:
## glm(formula = tetR ~ Sample + Day + offset(log(copydata_wide$"16S")),
##       family = "poisson", data = copydata_wide)
##
## Deviance Residuals:
##      Min       1Q    Median      3Q      Max
##  -463.7   -264.4    162.9    351.0    990.1
##
## Coefficients:
##                 Estimate  Std. Error  z value  Pr(>|z|)
## (Intercept) -1.8590168  0.0015652 -1187.7   <2e-16 ***
## SampleN       4.0260092  0.0015219  2645.3   <2e-16 ***
## Day           0.1573134  0.0005051   311.5   <2e-16 ***
## ---
## Signif. codes:  0 '***' 0.001 '**' 0.01 '*' 0.05 '.' 0.1 ' ' 1
##
## (Dispersion parameter for poisson family taken to be 1)
##
##   Null deviance: 6549438  on 11  degrees of freedom
## Residual deviance: 2070202 on 9 degrees of freedom
## AIC: 2070373
##
## Number of Fisher Scoring iterations: 6
```

```r
#"Estimate" column contains model coefficients in log form - as are
#other variables. Actual estimates/values are calculated later.

#Generating a model with 1Days1  as the only factor for predicting copy
#number ratio. This is used to check that "Sample" (i.e. KD vs. N)
#is main predictor of copy number ratio.
m2 <- glm(tetR ~ Day + offset(log(copydata_wide$"16S"))
              , data = copydata_wide
              , family = "poisson"
              )
summary(m2)
```

```
##
## Call:
## glm(formula = tetR ~ Day + offset(log(copydata_wide$"16S")),
##       family = "poisson", data = copydata_wide)
##
## Deviance Residuals:
```

```
##       Min        1Q    Median       3Q      Max
## -741.22     39.21   393.73   888.06  1149.72
##
## Coefficients:
##                 Estimate Std. Error z value Pr(>|z|)
## (Intercept) -2.0097883  0.0015647 -1284.5   <2e-16 ***
## Day          0.3477793  0.0004724   736.1   <2e-16 ***
## ---
## Signif. codes:  0 '***' 0.001 '**' 0.01 '*' 0.05 '.' 0.1 ' ' 1
##
## (Dispersion parameter for poisson family taken to be 1)
##
##     Null deviance: 6549438  on 11  degrees of freedom
## Residual deviance: 6081455  on 10  degrees of freedom
## AIC: 6081624
##
## Number of Fisher Scoring iterations: 7
```

```
#Checking dispersion of Sample + Day Poisson model
#Standard
with(m1, cbind(res.deviance = deviance, df = df.residual,
               p = pchisq(deviance, df.residual, lower.tail=FALSE)))
```

```
##      res.deviance df p
## [1,]      2070202  9 0
```

```
#using residual deviance to check goodness of fit. Insignificant p-values indicate
#that the residual difference is small enough and the model fits well (not true
#here).

#From 1performance1  package - checks Poisson models for over-dispersion.
check_overdispersion(m1)
```

```
## # Overdispersion test
##
##        dispersion ratio =  1427469.481
##   Pearson's Chi-Squared = 12847225.332
##                 p-value =  < 0.001
```

```
## Overdispersion detected.
```

```
#From 1DHARMa1  package
simulationOutput <- simulateResiduals(fittedModel = m1)
plot(simulationOutput)
```

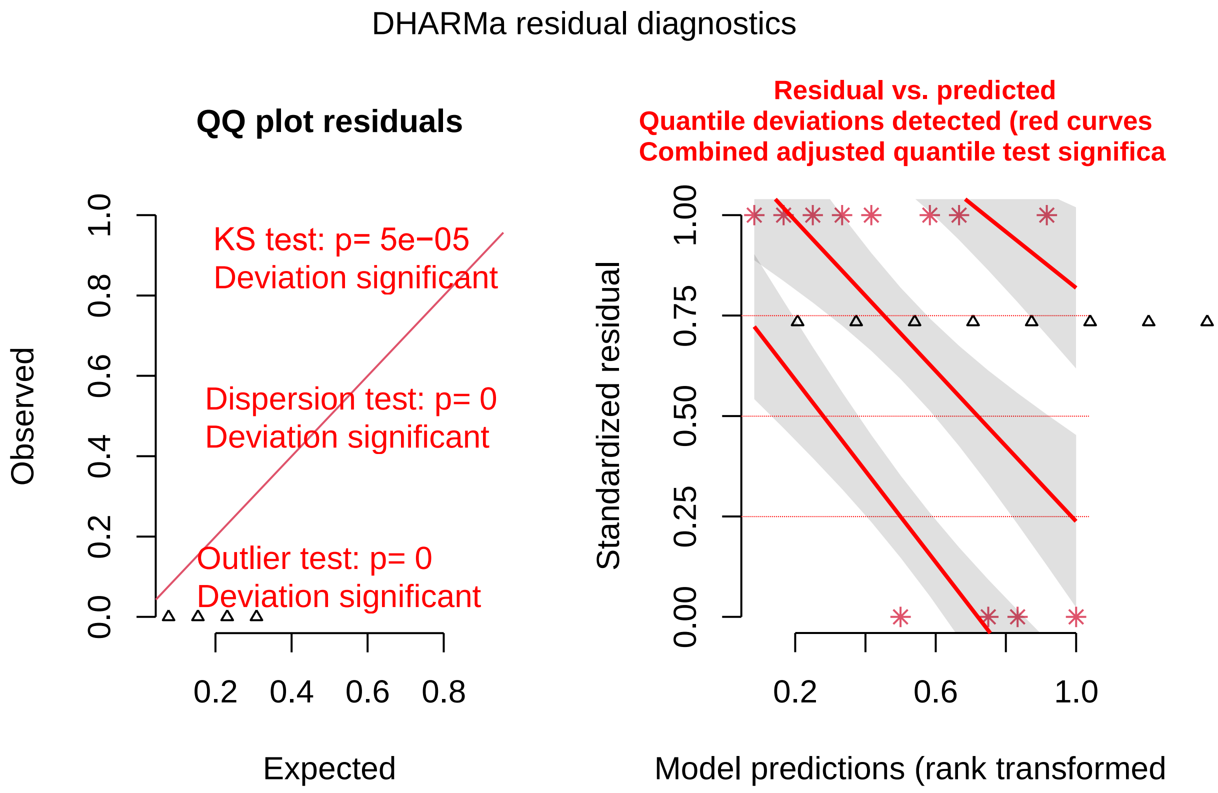


```
#this shows visually the over-dispersion of the Poisson model

#model appears to be over-dispersed - try quasi-Poisson distribution
m3 <- glm(tetR ~ Sample + Day
          + offset(log(copydata_wide$"16S"))
          , data = copydata_wide
          , family = "quasipoisson"
          )
summary(m3)
```

```
##
## Call:
## glm(formula = tetR ~ Sample + Day + offset(log(copydata_wide$"16S")),
##     family = "quasipoisson", data = copydata_wide)
##
## Deviance Residuals:
##     Min      1Q  Median      3Q     Max
##  -463.7  -264.4   162.9   351.0   990.1
##
## Coefficients:
##             Estimate Std. Error t value Pr(>|t|)
## (Intercept)  -1.8590     1.8700  -0.994   0.3461
## SampleN       4.0260     1.8184   2.214   0.0541 .
## Day           0.1573     0.6035   0.261   0.8002
## ---
## Signif. codes:  0 '***' 0.001 '**' 0.01 '*' 0.05 '.' 0.1 ' ' 1
```

```
##
## (Dispersion parameter for quasipoisson family taken to be 1427473)
##
##   Null deviance: 6549438  on 11  degrees of freedom
## Residual deviance: 2070202 on 9 degrees of freedom
## AIC: NA
##
## Number of Fisher Scoring iterations: 6
```

```
anova(m3, m1, test="Chisq")
```

```
## Analysis of Deviance Table
##
## Model 1: tetR ~ Sample + Day + offset(log(copydata_wide$"16S"))
## Model 2: tetR ~ Sample + Day + offset(log(copydata_wide$"16S"))
##  Resid. Df Resid. Dev Df Deviance Pr(>Chi)
## 1         9   2070202
## 2         9   2070202  0        0
```

```
with(m3, cbind(res.deviance = deviance, df = df.residual,
               p = pchisq(deviance, df.residual, lower.tail=FALSE)))
```

```
##      res.deviance df p
## [1,]      2070202  9 0
```

```
#ineffective, similarly high dispersion... take original Poisson model
#for ratio estimate, but move on to negative binomial regression - they
#tend to deal with over-dispersed data better.

#Generating actual estimates.
#Uses 1sandwich1 package to calculate robust SEs, which controls for
#violation of Poisson assumption - mean = var(Cameron and Trivedi, 2009)
cov.m1 <- vcovCR(m1, type="CR2", cluster = copydata_wide$Comparison)
std.err <- sqrt(diag(cov.m1))
pr.est <- cbind(Estimate= coef(m1),
                "Robust SE" = std.err,
                "Pr(>|z|)" = 2 * pnorm(abs(coef(m1)/std.err), lower.tail=FALSE),
                LL = coef(m1) - 1.96 * std.err,
                UL = coef(m1) + 1.96 * std.err)
pr.est
```

```
##               Estimate  Robust SE     Pr(>|z|)           LL         UL
## (Intercept) -1.8590168 0.36799672 4.378466e-07 -2.580290373 -1.1377432
## SampleN      4.0260092 0.25796503 6.543581e-55  3.520397708  4.5316206
## Day          0.1573134 0.08345309 5.942283e-02 -0.006254653  0.3208815
```

```
#uses 1msm1 package to convert rohbust SEs.
s <- deltamethod(list(~ 1/exp(x1), ~ 1/exp(x2), ~ 1/exp(x3)), coef(m1), cov.m1)
prexp.est <- 1/exp(pr.est[, -3])
prexp.est[, "Robust SE"] <- s
prexp.est <- cbind(prexp.est,"Pr(>|z|)" = pr.est[, 3])
prexp.est
```

```
##               Estimate   Robust SE          LL         UL     Pr(>|z|)
## (Intercept) 6.41742408 2.361591008 13.20097081 3.11971993 4.378466e-07
## SampleN     0.01784541 0.004603491  0.02958767 0.01076322 6.543581e-55
## Day         0.85443623 0.071305345  1.00627425 0.72550924 5.942283e-02
```

Generalised linear model (negative binomial)

```
#In this case, regression is sensitive to Bioreplicate = 1,
#Sample = KD, Day = 5 data point (which appears to be an outlier).
#Providing CorCopy data [i.e. C#(tetR)/(C#(16S)/sum(C#(16S))),
#where C# = copy number] allows the regression to solve, while
#multiplying by constant, sum(C#(16S)), which preserves the difference #in
means (i.e. KD/N transformed => regression 1Estimate1).
#See comparison of working regressions later (next section).
#Typical analysis follows that of Poisson, except where noted.

#generate negative binomial model
m4 <- glm.nb(CorCopy ~ Sample + Day
             , data = copydata_wide
             )
summary(m4)
```

```
##
## Call:
## glm.nb(formula = CorCopy ~ Sample + Day, data = copydata_wide,
##     init.theta = 0.5828682129, link = log)
##
## Deviance Residuals:
##     Min       1Q   Median       3Q      Max
## -1.8050  -1.1635  -0.6739  -0.2024   1.6260
##
## Coefficients:
##             Estimate Std. Error z value Pr(>|z|)
## (Intercept)  14.1231     1.0317  13.690  < 2e-16 ***
## SampleN       2.9629     0.7562   3.918 8.93e-05 ***
## Day           0.6688     0.2521   2.653  0.00798 **
## ---
## Signif. codes:  0 '***' 0.001 '**' 0.01 '*' 0.05 '.' 0.1 ' ' 1
##
## (Dispersion parameter for Negative Binomial(0.5829) family taken to be 1)
##
##     Null deviance: 24.360  on 11  degrees of freedom
## Residual deviance: 14.893  on  9  degrees of freedom
## AIC: 459.77
##
## Number of Fisher Scoring iterations: 3
##
##
##               Theta:  0.583
##           Std. Err.:  0.199
##
##  2 x log-likelihood:  -451.772
```

```
with(m4, cbind(res.deviance = deviance, df = df.residual,
  p = pchisq(deviance, df.residual, lower.tail=FALSE)))
```

```
##      res.deviance df          p
## [1,]     14.89283  9 0.09392205
```

```r
#residuals and deviance are much better than Poisson model.

m5 <- glm.nb(CorCopy ~ Day, data = copydata_wide)
anova(m5, m4, test="Chisq")
```

```
## Likelihood ratio tests of Negative Binomial Models
##
## Response: CorCopy
##              Model     theta Resid. df    2 x log-lik.   Test    df LR stat.
## 1              Day 0.3909587        10       -458.8650
## 2 Sample + Day 0.5828682         9       -451.7722 1 vs 2     1 7.092813
##        Pr(Chi)
## 1
## 2 0.007739365
```

```r
#significant p-value indicates that 1Sample1 is an important
#predictor of copy number ratio.

m4_d <- glm.nb(CorCopy ~ Sample, data = copydata_wide)
anova(m4_d, m4, test="Chisq")
```

```
## Likelihood ratio tests of Negative Binomial Models
##
## Response: CorCopy
##              Model     theta Resid. df    2 x log-lik.   Test    df LR stat.
## 1           Sample 0.4721122        10       -455.4502
## 2 Sample + Day 0.5828682         9       -451.7722 1 vs 2     1 3.678028
##       Pr(Chi)
## 1
## 2  0.05513401
```

```r
#1Day1 is not an important predictor for copy number ratio.

#model without day can also solve with using 1Corrected1 ratio data
m4_dc <- glm.nb(tetR ~ Sample + offset(log(copydata_wide$"16S")),
                data = copydata_wide)
summary(m4_dc)
```

```
##
## Call:
## glm.nb(formula = tetR ~ Sample + offset(log(copydata_wide$"16S")),
##     data = copydata_wide, init.theta = 0.4721137342, link = log)
##
## Deviance Residuals:
##     Min       1Q   Median       3Q      Max
## -1.6232  -1.2702  -1.0013   0.1192   1.6714
##
## Coefficients:
##             Estimate Std. Error z value Pr(>|z|)
## (Intercept)   1.6084     0.5942   2.707  0.00679 **
## SampleN       1.8665     0.8403   2.221  0.02633 *
## ---
## Signif. codes:  0 '***' 0.001 '**' 0.01 '*' 0.05 '.' 0.1 ' ' 1
##
## (Dispersion parameter for Negative Binomial(0.4721) family taken to be 1)
```

```
##
##     Null deviance: 19.731  on 11  degrees of freedom
## Residual deviance: 15.382  on 10  degrees of freedom
## AIC: 341.08
##
## Number of Fisher Scoring iterations: 1
##
##
##               Theta:  0.472
##           Std. Err.:  0.158
##
##  2 x log-likelihood:  -335.084
```

```r
#Relatively similar to m4 (using CorCopy, with 1Day1 as X variable), however,
#a solution that uses original data is far simpler and probably more robust.

#Modeling random effects associated with biological replicates within sample type
glme <- glmer.nb(tetR ~ Sample + Day + (1 | Comparison2) + offset(log(copydata_wide$"16S"))
             , data = copydata_wide)
```

```
## Warning in checkConv(attr(opt, "derivs"), opt$par, ctrl = control$checkConv, : Model is nearly unide
##  - Rescale variables?;Model is nearly unidentifiable: large eigenvalue ratio
##  - Rescale variables?
```

```r
summary(glme)
```

```
## Generalized linear mixed model fit by maximum likelihood (Laplace
##   Approximation) [glmerMod]
##  Family: Negative Binomial(0.8102)  ( log )
## Formula:
## tetR ~ Sample + Day + (1 | Comparison2) + offset(log(copydata_wide$"16S"))
##    Data: copydata_wide
##
##      AIC      BIC   logLik deviance df.resid
##    341.0    343.4   -165.5    331.0        7
##
## Scaled residuals:
##      Min       1Q   Median       3Q      Max
## -0.85791 -0.61263 -0.29520  0.07175  [illegible]
##
## Random effects:
##  Groups      Name        Variance Std.Dev.
##  Comparison2 (Intercept) 0.8165   0.9036
## Number of obs: 12, groups:  Comparison2, 6
##
## Fixed effects:
##             Estimate Std. Error z value Pr(>|z|)
## (Intercept)  -1.8466     0.8777  -2.104 0.035394 *
## SampleN       3.4173     0.9199   3.715 0.000203 ***
## Day           0.5365     0.2364   2.269 0.023249 *
## ---
## Signif. codes:  0 '***' 0.001 '**' 0.01 '*' 0.05 '.' 0.1 ' ' 1
##
## Correlation of Fixed Effects:
##         (Intr) SamplN
```

```
## SampleN -0.429
## Day      -0.653  -0.120
```

```
stargazer(glme,type = "text",
          digits  = 3,
          star.cutoffs = c(0.05, 0.01, 0.001),
          digit.separator = "")
```

```
##
## ===================================================
##                              Dependent variable:
##                          ---------------------------
##                                     tetR
## ---------------------------------------------------
## SampleN                          3.417***
##                                   (0.920)
##
## Day                               0.536*
##                                   (0.236)
##
## Constant                          -1.847*
##                                   (0.878)
##
## ---------------------------------------------------
## Observations                        12
## Log Likelihood                  -165.500
## Akaike Inf. Crit.                341.000
## Bayesian Inf. Crit.             343.424
## ===================================================
## Note:                 *p<0.05; **p<0.01; ***p<0.001
```

```
cov.m1 <- vcov.glmerMod(glme)
std.err <- sqrt(diag(cov.m1))
coefm <- as.numeric(coef(glme)$Comparison2[1,])
coefm[1] <- mean(as.numeric(coef(glme)$Comparison2[,1]))
glme.est <- cbind(Estimate= coefm,
                  "Robust SE" = std.err,
                  "Pr(>|z|)" = 2 * pnorm(abs(coefm/std.err), lower.tail=FALSE),
                  LL = coefm - 1.96 * std.err,
                  UL = coefm + 1.96 *std.err)

s <- deltamethod(list(~ exp(x1), ~ 1/exp(x2), ~ exp(x3)), coefm, cov.m1)
glmeexp.est <- exp(glme.est[,-3])
glmeexp.est[2,]  <-  1/exp(glme.est[2, -3])
glmeexp.est[, "Robust SE"] <-  s
glmeexp.est <- cbind(glmeexp.est,"Pr(>|z|)" = glme.est[, 3])
rownames(glmeexp.est) <- rownames(prexp.est)
glmeexp.est
```

```
##              Estimate  Robust SE         LL          UL     Pr(>|z|)
## (Intercept) 0.15776391 0.13847621 0.02823953 0.881369055 0.0353899957
## SampleN     0.03280226 0.03017404 0.19903011 0.005406157 0.0002032779
## Day         1.70996305 0.40423911 1.07586323 2.717793049 0.0232490264
```

```
#Generating actual estimates. As not Poisson model, robust
#SEs are not theoretically required. However, 1confint()1
```

```r
#cannot converge, so used robust SE method again.
#1confint()1  converges once outlier (in KD day 5) removed.
#e.g. cbind(Estimate = 1/exp(coef(m4)),  1/exp(confint(m4)))
cov.m1 <- vcovCR(m4, type="CR2", cluster = copydata_wide$Comparison) #"CR2" recommended by  authors
std.err <-  sqrt(diag(cov.m1))
nbr.est <- cbind(Estimate= coef(m4),
                 "Robust SE" = std.err,
                 "Pr(>|z|)" = 2 * pnorm(abs(coef(m4)/std.err), lower.tail=FALSE),
                 LL = coef(m4) - 1.96 * std.err,
                 UL = coef(m4) + 1.96 *  std.err)

s <- deltamethod(list(~ exp(x1)/6560166, ~ 1/exp(x2), ~ exp(x3)), coef(m4), cov.m1)
nbrexp.est <- exp(nbr.est[, -3])
nbrexp.est[1,] <- nbrexp.est[1,]/6560166 #16S copy number sum
nbrexp.est[2,] <- 1/exp(nbr.est[2, -3])
nbrexp.est[, "Robust SE"] <- s
nbrexp.est <- cbind(nbrexp.est,"Pr(>|z|)" = nbr.est[, 3])
nbrexp.est
```

```
##                 Estimate  Robust SE         LL          UL     Pr(>|z|)
## (Intercept) 0.20732749 0.37014199 0.00626582 6.860185355 2.558426e-15
## SampleN     0.05166688 0.06761415 0.67167972 0.003974315 2.356742e-02
## Day         1.95185063 0.85143330 0.83009905 4.589477458 1.252448e-01
```

Negative binomial model without outlier

```
#Removing outlier KD day 5 BR1 from data and repeating negative binomial model
#See 1Poisson1 section for line-by-line description of code as analysis
#follows the same procedure.

#Generating new negative binomial model with transformed copy number ratio
m6 <- glm.nb(CorCopy ~ Sample + Day
             , data =copydata_wide_KD
             )
summary(m6)
```

```
##
## Call:
## glm.nb(formula = CorCopy ~ Sample + Day, data = copydata_wide_KD,
##     init.theta = 1.584596635, link = log)
##
## Deviance Residuals:
##     Min       1Q   Median       3Q      Max
## -1.8924  -0.9933  -0.1583   0.4911   1.1027
##
## Coefficients:
##             Estimate Std. Error z value Pr(>|z|)
## (Intercept)  14.1214     0.6262  22.550   <2e-16 ***
## SampleN       4.6226     0.4835   9.562   <2e-16 ***
## Day           0.1156     0.1612   0.717    0.473
## ---
## Signif. codes:  0 '***' 0.001 '**' 0.01 '*' 0.05 '.' 0.1 ' ' 1
##
## (Dispersion parameter for Negative Binomial(1.5846) family taken to be 1)
##
##     Null deviance: 65.857  on 10  degrees of freedom
## Residual deviance: 12.117  on  8  degrees of freedom
## AIC: 403.46
##
## Number of Fisher Scoring iterations: 1
##
##
##               Theta:  1.585
##           Std. Err.:  0.618
##
##  2 x log-likelihood:  -395.458
```

```
with(m6, cbind(res.deviance = deviance, df = df.residual,
  p = pchisq(deviance, df.residual, lower.tail=FALSE)))
```

```
##      res.deviance df         p
## [1,]     12.11735  8 0.1460442
```

```
#Notable improvement in residual (makes)

#Generating negative binomial model with copy number ratio
m7 <- glm.nb(tetR ~ Sample + Day
                 + offset(log(copydata_wide_KD$"16S")),
                 data = copydata_wide_KD
```

```r
        )
summary(m7)
```

```
##
## Call:
## glm.nb(formula = tetR ~ Sample + Day + offset(log(copydata_wide_KD$"16S")),
##     data = copydata_wide_KD, init.theta = 1.584615156, link = log)
##
## Deviance Residuals:
##     Min       1Q   Median       3Q      Max
## -1.8924  -0.9933  -0.1583   0.4911   1.1027
##
## Coefficients:
##             Estimate Std. Error z value Pr(>|z|)
## (Intercept) -1.5752 0.6262 -2.515 0.0119 *
## SampleN      4.6226     0.4835   9.562   <2e-16 ***
## Day          0.1156     0.1612   0.717   0.4734
## ---
## Signif. codes:  0 '***' 0.001 '**' 0.01 '*' 0.05 '.' 0.1 ' ' 1
##
## (Dispersion parameter for Negative Binomial(1.5846) family taken to be 1)
##
##     Null deviance: 65.857  on 10  degrees of freedom
## Residual deviance: 12.117  on  8  degrees of freedom
## AIC: 297.44
##
## Number of Fisher Scoring iterations: 1
##
##
##               Theta:  1.585
##           Std. Err.:  0.618
##
##  2 x log-likelihood:  -289.444
```

```r
with(m7, cbind(res.deviance = deviance, df = df.residual,
  p = pchisq(deviance, df.residual, lower.tail=FALSE)))
```

```
##      res.deviance df         p
## [1,]     12.11734  8 0.1460447
```

```r
#residuals slightly better than transformed data
m7d <- glm.nb(tetR ~ Day
             + offset(log(copydata_wide_KD$"16S")),
             data = copydata_wide_KD)
#model does not converge fully
anova(m7d,m7, test="Chisq")
```

```
## Likelihood ratio tests of Negative Binomial Models
##
## Response: tetR
##                                                   Model      theta Resid. df
## 1          Day  + offset(log(copydata_wide_KD$"16S")) 0.02290644         9
## 2 Sample + Day + offset(log(copydata_wide_KD$"16S")) 1.58461516         8
##     2 x log-lik.   Test    df LR stat.      Pr(Chi)
## 1      -353.9999
```

```
## 2          -289.4444 1 vs 2       1 64.55542 8.881784e-16
```

```r
m7b <- glm.nb(tetR ~ Sample + Day + Bioreplicate
              + offset(log(copydata_wide_KD$"16S")),
              data = copydata_wide_KD)
anova(m7b,m7, test="Chisq")
```

```
## Likelihood ratio tests of Negative Binomial Models
##
## Response: tetR
##                                                              Model    theta
## 1                Sample + Day + offset(log(copydata_wide_KD$"16S")) 1.584615
## 2 Sample + Day + Bioreplicate + offset(log(copydata_wide_KD$"16S")) 1.810408
## Resid. df 2 x log-lik. Test df LR stat.  Pr(Chi)
## 1         8      -289.4444
## 2         6      -287.7072 1 vs 2 2 1.737222 0.4195338
```

```r
#1Sample1 is still important predictor of copy number ratio,
#also effect of 1Bioreplicate1  is not significant.

#model without 1Day1
m7_d <- glm.nb(tetR ~ Sample
              + offset(log(copydata_wide_KD$"16S")),
              data = copydata_wide_KD)
summary(m7_d)
```

```
##
## Call:
## glm.nb(formula = tetR ~ Sample + offset(log(copydata_wide_KD$"16S")),
##     data = copydata_wide_KD, init.theta = 1.525671133, link = log)
##
## Deviance Residuals:
##      Min        1Q    Median        3Q       Max
## -1.85176  -0.94927   0.02713   0.50784   1.31403
##
## Coefficients:
##             Estimate Std. Error z value Pr(>|z|)
## (Intercept)  -1.2008     0.3621  -3.317 0.000911 ***
## SampleN       4.6756     0.4902   9.537  < 2e-16 ***
## ---
## Signif. codes:  0 '***' 0.001 '**' 0.01 '*' 0.05 '.' 0.1 ' ' 1
##
## (Dispersion parameter for Negative Binomial(1.5257) family taken to be 1)
##
##     Null deviance: 63.407  on 10  degrees of freedom
## Residual deviance: 12.158  on  9  degrees of freedom
## AIC: 295.94
##
## Number of Fisher Scoring iterations: 1
##
##
##               Theta:  1.526
##           Std. Err.:  0.593
##
##  2 x log-likelihood:  -289.945
```

```r
with(m7_d, cbind(res.deviance = deviance, df = df.residual,
  p = pchisq(deviance, df.residual, lower.tail=FALSE)))
```

```
##      res.deviance df         p
## [1,]      12.1577  9 0.2045641
```

```r
anova(m7_d,m7, test="Chisq")
```

```
## Likelihood ratio tests of Negative Binomial Models
##
## Response: tetR
##                                                   Model    theta Resid. df
## 1          Sample + offset(log(copydata_wide_KD$"16S")) 1.525671        9
## 2  Sample + Day + offset(log(copydata_wide_KD$"16S")) 1.584615        8
##      2 x log-lik.   Test    df LR stat.  Pr(Chi)
## 1      -289.9449
## 2      -289.4444 1 vs 2      1 0.500451 0.479302
```

```r
#1Day1 is not important predictor of copy number ratio.

#Poisson model for comparison
m7p <- glm(tetR ~ Sample + Day + offset(log(copydata_wide_KD$"16S"))
          , data = copydata_wide_KD
          , family = "poisson"
          )
summary(m7p)
```

```
##
## Call:
## glm(formula = tetR ~ Sample + Day + offset(log(copydata_wide_KD$"16S")),
##     family = "poisson", data = copydata_wide_KD)
##
## Deviance Residuals:
##    Min      1Q  Median      3Q     Max
## -407.1  -245.6   173.9   315.7   394.9
##
## Coefficients:
##              Estimate Std. Error z value Pr(>|z|)
## (Intercept) -1.7159526  0.0016092 -1066.3   <2e-16 ***
## SampleN      4.2204351  0.0015440  2733.5   <2e-16 ***
## Day          0.0638088  0.0005424   117.7   <2e-16 ***
## ---
## Signif. codes:  0 '***' 0.001 '**' 0.01 '*' 0.05 '.' 0.1 ' ' 1
##
## (Dispersion parameter for poisson family taken to be 1)
##
##     Null deviance: 5560109  on 10  degrees of freedom
## Residual deviance: 1045655  on  8  degrees of freedom
## AIC: 1045812
##
## Number of Fisher Scoring iterations: 5
```

```r
#Generating estimates.
cov.m1 <- vcovCR(m7, type="CR2", cluster = copydata_wide_KD$Comparison)
std.err <- sqrt(diag(cov.m1))
nbr_KD.est <- cbind(Estimate= coef(m7),
```

```
                  "Robust SE" = std.err,
                  "Pr(>|z|)" = 2 * pnorm(abs(coef(m7)/std.err), lower.tail=FALSE),
                  LL = coef(m7) - 1.96 * std.err,
                  UL = coef(m7) + 1.96 * std.err)

s <- deltamethod(list(~ exp(x1), ~ 1/exp(x2), ~ exp(x3)), coef(m7), cov.m1)
nbrexp_KD.est <- exp(nbr_KD.est[, -3])
nbrexp_KD.est[2,] <- 1/exp(nbr_KD.est[2, -3])
nbrexp_KD.est[, "Robust SE"] <- s
nbrexp_KD.est <- cbind(nbrexp_KD.est,"Pr(>|z|)" = nbr_KD.est[, 3])

glme_KD <- glmer.nb(tetR ~ Sample + Day + (1 | Comparison2) + offset(log(copydata_wide_KD$"16S"))
              , data = copydata_wide_KD)
```

```
## boundary (singular) fit: see ?isSingular
```

```
summary(glme_KD)
```

```
## Generalized linear mixed model fit by maximum likelihood (Laplace
##   Approximation) [glmerMod]
##  Family: Negative Binomial(1.5846)  ( log )
## Formula:
## tetR ~ Sample + Day + (1 | Comparison2) + offset(log(copydata_wide_KD$"16S"))
##    Data: copydata_wide_KD
##
##      AIC      BIC   logLik deviance df.resid
##    299.4    301.4   -144.7    289.4        6
##
## Scaled residuals:
##     Min      1Q  Median      3Q     Max
## -1.0874 -0.7495 -0.1518  0.5576  [illegible]
##
## Random effects:
##  Groups      Name        Variance  Std.Dev.
##  Comparison2 (Intercept) 1.161e-13 3.408e-07
## Number of obs: 11, groups:  Comparison2, 6
##
## Fixed effects:
##             Estimate Std. Error z value Pr(>|z|)
## (Intercept)  -1.5752     0.5843  -2.696  0.00702 **
## SampleN       4.6226     0.4811   9.608  < 2e-16 ***
## Day           0.1156     0.1560   0.741  0.45890
## ---
## Signif. codes:  0 '***' 0.001 '**' 0.01 '*' 0.05 '.' 0.1 ' ' 1
##
## Correlation of Fixed Effects:
##         (Intr) SamplN
## SampleN -0.283
## Day     -0.803 -0.181
## convergence code: 0
## boundary (singular) fit: see ?isSingular
```

```
stargazer(glme_KD,type = "text",
          digits = 3,
          star.cutoffs = c(0.05, 0.01, 0.001),
```

```
                 digit.separator = "")
```

```
##
## ====================================================
##                                   Dependent variable:
##                              -------------------------------
##                                          tetR
##  -------------------------------------------------------
## SampleN                              4.623***
##                                       (0.481)
##
## Day                                   0.116
##                                       (0.156)
##
## Constant                             -1.575**
##                                       (0.584)
##
##  -------------------------------------------------------
## Observations                            11
## Log Likelihood                      -144.722
## Akaike Inf. Crit.                    299.444
## Bayesian Inf. Crit.                  301.434
## ====================================================
## Note:                     *p<0.05; **p<0.01; ***p<0.001
```

```
cov.m1 <- vcov.glmerMod(glme_KD)
std.err <- sqrt(diag(cov.m1))
coefm <- as.numeric(coef(glme_KD)$Comparison2[1,])
coefm[1] <- mean(as.numeric(coef(glme_KD)$Comparison2[,1]))
glme_KD.est <- cbind(Estimate= coefm,
                     "Robust SE" = std.err,
                     "Pr(>|z|)" = 2 * pnorm(abs(coefm/std.err), lower.tail=FALSE),
                     LL = coefm - 1.96 * std.err,
                     UL = coefm + 1.96 *std.err)

s <- deltamethod(list(~ exp(x1), ~ 1/exp(x2), ~ exp(x3)), coefm, cov.m1)
glmeexp_KD.est <- exp(glme_KD.est[, -3])
glmeexp_KD.est[2,] <- 1/exp(glme_KD.est[2, -3])
glmeexp_KD.est[, "Robust SE"] <- s
glmeexp_KD.est <- cbind(glmeexp_KD.est,"Pr(>|z|)" = glme_KD.est[, 3])
rownames(glmeexp_KD.est) <- rownames(prexp.est)
glmeexp_KD.est
```

```
##                Estimate   Robust SE         LL         UL     Pr(>|z|)
## (Intercept) 0.206970341 0.120929624 0.06584986 0.65052108 7.019643e-03
## SampleN     0.009827136 0.004728062 0.02523290 0.00382725 7.400519e-22
## Day         1.122494654 0.175124639 0.82676611 1.52400326 4.588980e-01
```

```
nbrexp_KD.est
```

```
##               Estimate   Robust SE         LL          UL     Pr(>|z|)
## (Intercept) 0.20697138 0.051458222 0.12713835 0.336933364 2.364974e-10
## SampleN     0.00982711 0.002412996 0.01590149 0.006073148 4.629811e-79
## Day         1.12249192 0.095021675 0.95087979 1.325076133 1.722509e-01
```

Linear models of log-transformed copy number ratio

```
#These models are used to compare R to SigmaPlot, as a software comparison of similar
#methods.
#As above, see 1Poisson1  section for line-by-line description of code as analysis
#follows the same procedure.

lm1 <- lm(logmean ~ Sample + Day, data = pointdata)
summary(lm1)
```

```
##
## Call:
## lm(formula = logmean ~ Sample + Day, data = pointdata)
##
## Residuals:
##     Min      1Q  Median      3Q     Max
## -1.9647 -0.9791  0.0245  0.4222  3.4387
##
## Coefficients:
##             Estimate Std. Error t value Pr(>|t|)
## (Intercept)  -1.8443     1.3136  -1.404  0.19386
## SampleN       3.5630     0.9629   3.700  0.00492 **
## Day           0.3602     0.3210   1.122  0.29073
## ---
## Signif. codes:  0 '***' 0.001 '**' 0.01 '*' 0.05 '.' 0.1 ' ' 1
##
## Residual standard error: 1.668 on 9 degrees of freedom
## Multiple R-squared:  0.6243, Adjusted R-squared:  0.5408
## F-statistic: 7.476 on 2 and 9 DF,  p-value: 0.01222
```

```
cbind(Estimate = exp(-coef(lm1)), exp(-confint(lm1)))
```

```
##              Estimate       2.5 %      97.5 %
## (Intercept) 6.3236784 123.4451603 0.323940670
## SampleN     0.0283548   0.2503731 0.003211186
## Day         0.6975042   1.4416745 0.337463229
```

```
lm1_d <- lm(logmean ~ Sample, data = pointdata)

cov.m1 <- vcovCR(lm1, type="CR2", cluster = pointdata$Comparison)
std.err <- sqrt(diag(cov.m1))
lm1r.est <- cbind(Estimate= coef(lm1),
                  "Robust SE" = std.err,
                  "Pr(>|z|)" = 2 * pnorm(abs(coef(lm1)/std.err), lower.tail=FALSE),
                  LL = coef(lm1) - 1.96 * std.err,
                  UL = coef(lm1) + 1.96 * std.err)

s <- deltamethod(list(~ exp(x1), ~ 1/exp(x2), ~ exp(x3)), coef(lm1), cov.m1)
lm1rexp.est <- exp(lm1r.est[, -3])
lm1rexp.est[2,] <- 1/exp(lm1r.est[2, -3])
lm1rexp.est[, "Robust SE"] <- s
lm1rexp.est <- cbind(lm1rexp.est,"Pr(>|z|)" = lm1r.est[, 3])
lm1rexp.est
```

```
##              Estimate Robust SE         LL          UL     Pr(>|z|)
## (Intercept) 0.1581358 0.1310285 0.03116928 0.802294296 2.602413e-02
```

```
## SampleN       0.0283548  0.0172217  0.09324505 0.008622384 4.457339e-09
## Day           1.4336831  0.2902561  0.96409557 2.131995209 7.517542e-02
```

```r
lme <- lmer(logmean ~ Sample + Day + (1 | Comparison2), data = pointdata)
summary(lme)
```

```
## Linear mixed model fit by REML ['lmerMod']
## Formula: logmean ~ Sample + Day + (1 | Comparison2)
##    Data: pointdata
##
## REML criterion at convergence: 41.6
##
## Scaled residuals:
##      Min       1Q   Median       3Q      Max
## -1.14808 -0.52145 -0.02798  0.20696  1.97440
##
## Random effects:
##  Groups      Name        Variance Std.Dev.
##  Comparison2 (Intercept) 0.2311   0.4807
##  Residual                2.5760   1.6050
## Number of obs: 12, groups:  Comparison2, 6
##
## Fixed effects:
##             Estimate Std. Error t value
## (Intercept)  -1.8443     1.2942  -1.425
## SampleN       3.5630     1.0063   3.541
## Day           0.3602     0.3089   1.166
```

```r
stargazer(lme,type = "text",
          digits = 3,
          star.cutoffs = c(0.05, 0.01, 0.001),
          digit.separator = "")
```

```
##
## ==================================================
##                          Dependent variable:
##                     -----------------------------
##                                logmean
## --------------------------------------------------
## SampleN                       3.563***
##                                (1.006)
##
## Day                             0.360
##                                (0.309)
##
## Constant                       -1.844
##                                (1.294)
##
## --------------------------------------------------
## Observations                     12
## Log Likelihood                -20.798
## Akaike Inf. Crit.              51.596
## Bayesian Inf. Crit.            54.021
## ==================================================
## Note:                *p<0.05; **p<0.01; ***p<0.001
```

```
cov.m1 <- vcovCR(lme, type="CR2")
std.err <- sqrt(diag(cov.m1))
coefm <- as.numeric(coef(lme)$Comparison2[1,])
coefm[1] <- mean(as.numeric(coef(lme)$Comparison2[,1]))
lme.est <- cbind(Estimate= coefm,
                 "Robust SE" = std.err,
                 "Pr(>|z|)" = 2 * pnorm(abs(coefm/std.err), lower.tail=FALSE),
                 LL = coefm - 1.96 * std.err,
                 UL = coefm + 1.96 *std.err)

s <- deltamethod(list(~ exp(x1), ~ 1/exp(x2), ~ exp(x3)), coefm, cov.m1)
lmeexp.est <- exp(lme.est[,-3])
lmeexp.est[2,] <- 1/exp(lme.est[2, -3])
lmeexp.est[, "Robust SE"] <- s
lmeexp.est <- cbind(lmeexp.est,"Pr(>|z|)" = lme.est[, 3])
lmeexp.est
```

```
##              Estimate  Robust SE         LL          UL     Pr(>|z|)
## (Intercept) 0.1581358 0.14051272 0.02771246 0.902371573 0.0379297442
## SampleN     0.0283548 0.02853413 0.20381078 0.003944809 0.0003992679
## Day         1.4336831 0.44283341 0.78258360 2.626488895 0.2434904402
```

```
lm2 <- lm(logmean ~ Sample + Day, data = pointdata_KD)
summary(lm2)
```

```
##
## Call:
## lm(formula = logmean ~ Sample + Day, data = pointdata_KD)
##
## Residuals:
##     Min      1Q  Median      3Q     Max
## -1.4794 -0.7435  0.4010  0.5374  1.3136
##
## Coefficients:
##             Estimate Std. Error t value Pr(>|t|)
## (Intercept) -1.7169 0.8484 -2.024 0.077596 .
## SampleN      4.3271     0.6550   6.607 0.000168 ***
## Day          0.1055     0.2183   0.483 0.641789
## ---
## Signif. codes:  0 '***' 0.001 '**' 0.01 '*' 0.05 '.' 0.1 ' ' 1
##
## Residual standard error: 1.076 on 8 degrees of freedom
## Multiple R-squared:  0.849,  Adjusted R-squared:  0.8112
## F-statistic: 22.49 on 2 and 8 DF,  p-value: 0.0005203
```

```
cbind(Estimate = exp(-coef(lm2)), exp(-confint(lm2)))
```

```
##               Estimate       2.5 %      97.5 %
## (Intercept) 5.56747414 39.38026912 0.787114182
## SampleN     0.01320557  0.05979813 0.002916264
## Day         0.89984969  1.48872140 0.543909330
```

```
lm2_d <- lm(logmean ~ Sample, data = pointdata_KD)
```

Calculating 95% CIs for comparison

```
#All R-related confidence intervals in Supplementary Table S11 are generated here.
#First with-outlier CIs are generated, then removal of outlier is justified and
#visualised, before without-outlier CIs are generated.

#lm(vcovCR)
cov.m1 <- vcovCR(lm1, type="CR2", cluster = pointdata$Comparison)
lm1ci <- exp(Confint(lm1, vcov. = cov.m1))
```

```
## Standard errors computed by cov.m1
```

```
lm1ci[2,] <- exp(-Confint(lm1, vcov. = cov.m1)[2,])
```

```
## Standard errors computed by cov.m1
```

```
lm1ci
```

```
##              Estimate      2.5 %      97.5 %
## (Intercept) 0.1581358 0.02426587 1.030539328
## SampleN     0.0283548 0.11202819 0.007176717
## Day         1.4336831 0.90688644 2.266487892
```

```
#lm
lm1ci1 <- exp(Confint(lm1))
lm1ci1[2,]  <-  exp(-Confint(lm1)[2,])
lm1ci1
```

```
##              Estimate       2.5 %      97.5 %
## (Intercept) 0.1581358 0.008100763 3.086985036
## SampleN     0.0283548 0.250373142 0.003211186
## Day         1.4336831 0.693637830 2.963285817
```

```
#lm_d
lm1ci1_d <- exp(Confint(lm1_d))
lm1ci1_d[2,]  <-  exp(-Confint(lm1_d)[2,])
lm1ci1_d
```

```
##              Estimate     2.5 %      97.5 %
## (Intercept) 0.5579770 0.1200260 2.593923672
## SampleN     0.0283548 0.2491088 0.003227483
```

```
#lme
cov.m1 <- vcovCR(lme, type="CR2")
lmeci <- exp(Confint(lme, vcov. = cov.m1))
lmeci[2,] <- exp(-Confint(lme, vcov. = cov.m1)[2,])
lmeci
```

```
##              Estimate      2.5 %      97.5 %
## (Intercept) 0.1581358 0.01251321 1.998442875
## SampleN     0.0283548 0.20380338 0.003944953
## Day         1.4336831 0.78259229 2.626459737
```

```
#aov
lnm <- aov(logmean ~ Sample + Day, data = pointdata)
lnmci <- exp(Confint(lnm))
lnmci[2,]  <-  exp(-Confint(lnm)[2,])
lnmci
```

```
##              Estimate       2.5  %       97.5  %
```

```
## (Intercept) 0.1581358  0.008100763  3.086985036
## SampleN      0.0283548  0.250373142  0.003211186
## Day          1.4336831  0.693637830  2.963285817
```

```
anova(lm1, lnm) #aov produces essentially the same result as lm
```

```
## Analysis of Variance Table
##
## Model 1: logmean ~ Sample + Day
## Model 2: logmean ~ Sample + Day
##   Res.Df    RSS Df Sum of Sq F Pr(>F)
## 1      9 25.032
## 2      9 25.032  0          0
```

```
#glm.nb(vcovCR)
cov.m1 <- vcovCR(m4, type="CR2", cluster = copydata_wide$Comparison)
glmci <- exp(Confint(m4, vcov. = cov.m1))
```

```
## Standard errors computed by cov.m1
```

```
glmci[1,] <- exp(Confint(m4, vcov. = cov.m1)[1,])/6560166
```

```
## Standard errors computed by cov.m1
```

```
glmci[2,] <- exp(-Confint(m4, vcov. = cov.m1)[2,])
```

```
## Standard errors computed by cov.m1
```

```
glmci
```

```
##                Estimate      2.5 %     97.5 %
## (Intercept) 0.20732749 0.02744724 1.56608408
## SampleN     0.05166688 0.22746618 0.01173566
## Day         1.95185063 1.19090848 3.19900391
```

```
#glm.nb
glmci1 <- exp(Confint(m4))
```

```
## Warning: glm.fit: algorithm did not converge
```

```
glmci1[1,] <- exp(Confint(m4)[1,])/6560166
```

```
## Warning: glm.fit: algorithm did not converge
```

```
glmci1[2,] <- exp(-Confint(m4)[2,])
```

```
## Warning: glm.fit: algorithm did not converge
```

```
glmci1
```

```
##                Estimate      2.5 %      97.5 %
## (Intercept) 0.20732749 0.01336037 5.438402019
## SampleN     0.05166688 0.36043728 0.007406189
## Day         1.95185063 1.02150563 3.729515426
```

```
#glm.nb_d
glmci1_d <- exp(Confint(m4_d))
```

```
## Warning: glm.fit: algorithm did not converge
```

```
glmci1_d[1,] <- exp(Confint(m4_d)[1,])/6560166
```

```
## Warning: glm.fit: algorithm did not converge
```

```r
glmci1_d[2,] <- exp(-Confint(m4_d)[2,])
```

```
## Warning: glm.fit: algorithm did not converge
```

```r
glmci1_d
```

```
##              Estimate     2.5 %      97.5 %
## (Intercept) 4.9947828 1.8822397 21.12022735
## SampleN     0.1546706 0.8826753  0.02710284
```

```r
#glm.nb_dc
glmci1_dc <- exp(Confint(m4_dc))
glmci1_dc[2,] <- exp(-Confint(m4_dc)[2,])
glmci1_dc
```

```
##              Estimate     2.5 %      97.5 %
## (Intercept) 4.9947098 1.8821863 21.12000324
## SampleN     0.1546689 0.8826707  0.02710237
```

```r
#glmer.nb
cov.m1 <- vcov.glmerMod(glme)
glmeci <- exp(Confint(glme, vcov. = cov.m1))
glmeci[2,] <- exp(-Confint(glme, vcov. = cov.m1)[2,])
glmeci
```

```
##               Estimate      2.5 %      97.5 %
## (Intercept) 0.15777096 0.02824169 0.881380556
## SampleN     0.03280226 0.19902352 0.005406337
## Day         1.70996305 1.07587239 2.717769910
```

```r
#justification for potential outlier (standardised residuals > 3)
plot(lm1$residuals)
```

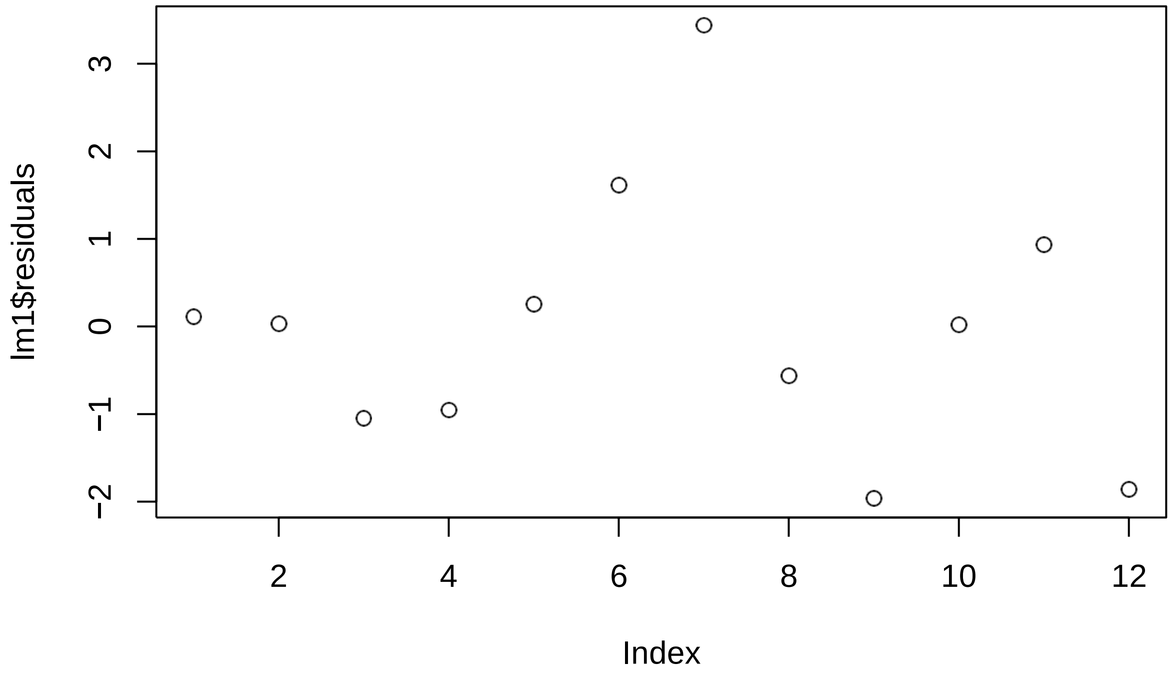


```
as.data.frame(abs(lm1$residuals)>3)
```

```
##       abs(lm1$residuals) > 3
## X1                     FALSE
## X2                     FALSE
## X3                     FALSE
## X4                     FALSE
## X5                     FALSE
## X6                     FALSE
## X7                      TRUE
## X8                     FALSE
## X9                     FALSE
## X10                    FALSE
## X11                    FALSE
## X12                    FALSE
```

```
plot(lm2$residuals)
```

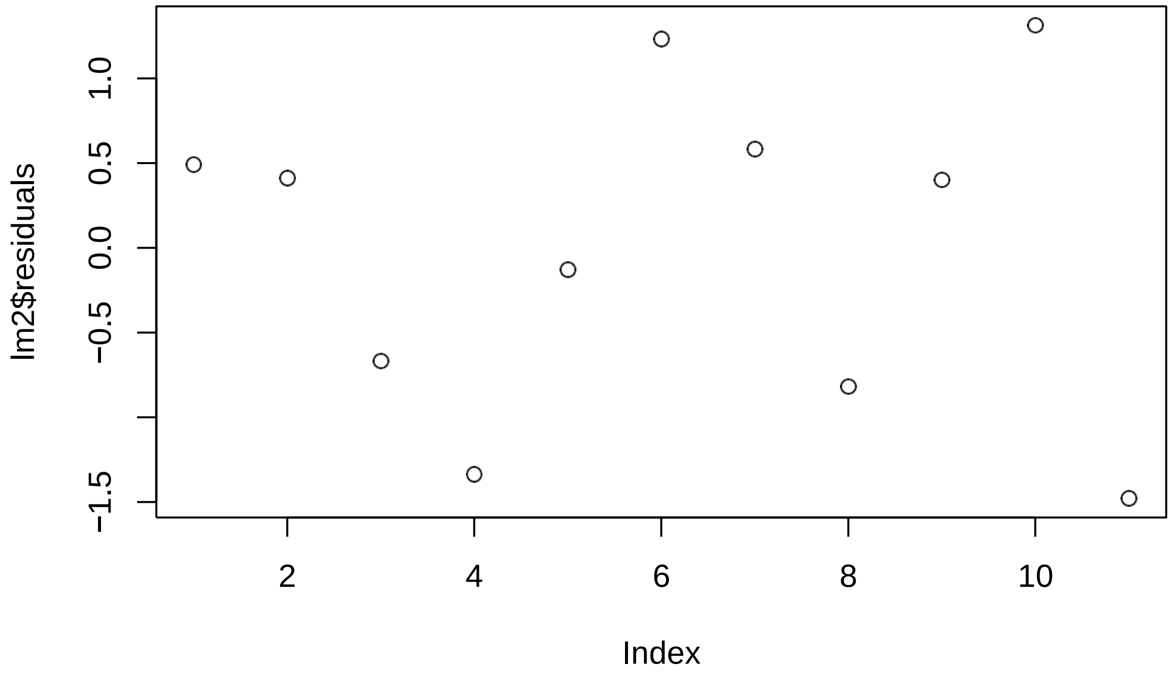


```
as.data.frame(abs(lm2$residuals)>3)
```

```
##       abs(lm2$residuals) > 3
## X1                     FALSE
## X2                     FALSE
## X3                     FALSE
## X4                     FALSE
## X5                     FALSE
## X6                     FALSE
## X8                     FALSE
## X9                     FALSE
## X10                    FALSE
## X11                    FALSE
## X12                    FALSE
```

```
#lm1 clearly have a potential outlier,  overall
#regression (lm2) improves after removal of outlier.
plot(m4$residuals)
```

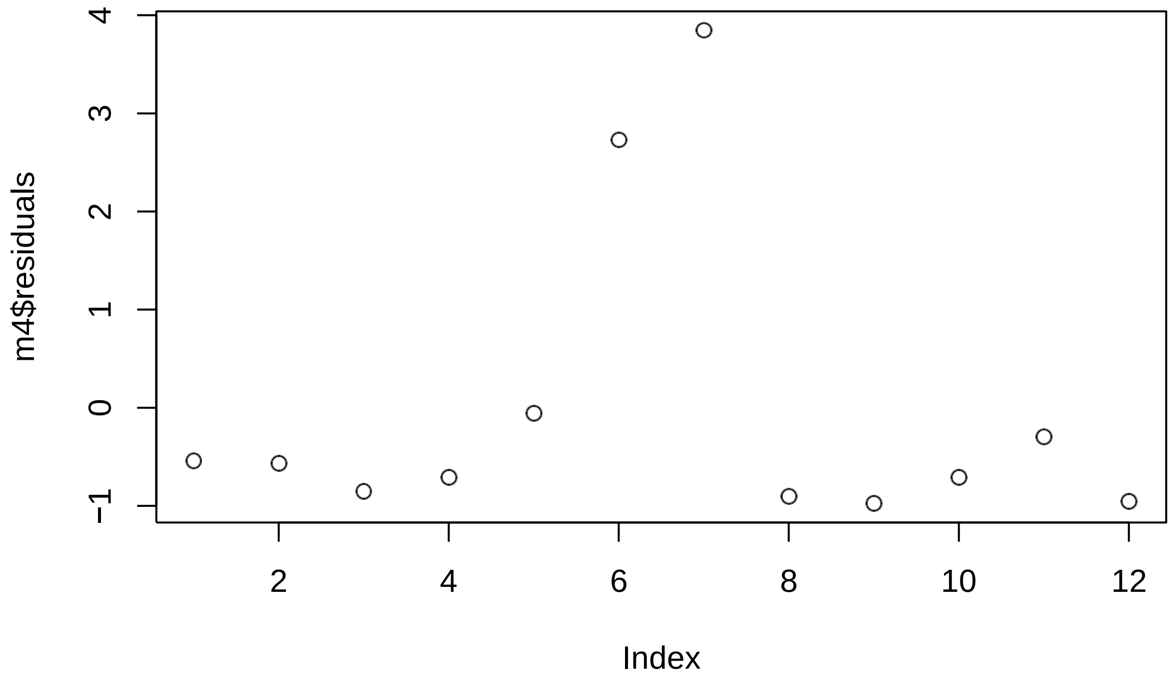


```r
as.data.frame(abs(m4$residuals)>3)
```

```
##    abs(m4$residuals) > 3
## 1                  FALSE
## 2                  FALSE
## 3                  FALSE
## 4                  FALSE
## 5                  FALSE
## 6                  FALSE
## 7                   TRUE
## 8                  FALSE
## 9                  FALSE
## 10                 FALSE
## 11                 FALSE
## 12                 FALSE
```

```r
as.data.frame(abs(m4_dc$residuals)>3)
```

```
##   abs(m4_dc$residuals) > 3
## 1                    FALSE
## 2                    FALSE
## 3                    FALSE
## 4                    FALSE
## 5                    FALSE
## 6                    FALSE
## 7                     TRUE
## 8                    FALSE
```

```
## 9                         FALSE
## 10                        FALSE
## 11                        FALSE
## 12                        FALSE
```

```
plot(m7$residuals)
```

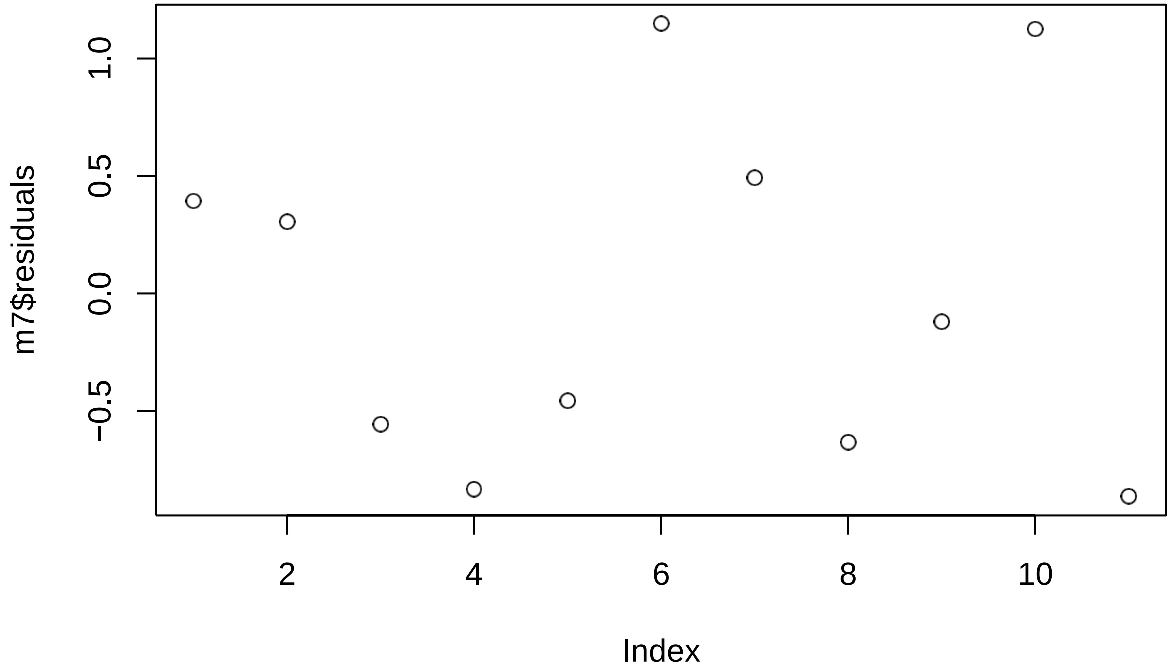


```
as.data.frame(abs(m7$residuals)>3)
```

```
##    abs(m7$residuals) > 3
## 1                  FALSE
## 2                  FALSE
## 3                  FALSE
## 4                  FALSE
## 5                  FALSE
## 6                  FALSE
## 8                  FALSE
## 9                  FALSE
## 10                 FALSE
## 11                 FALSE
## 12                 FALSE
```

```
plot(m7_d$residuals)
```

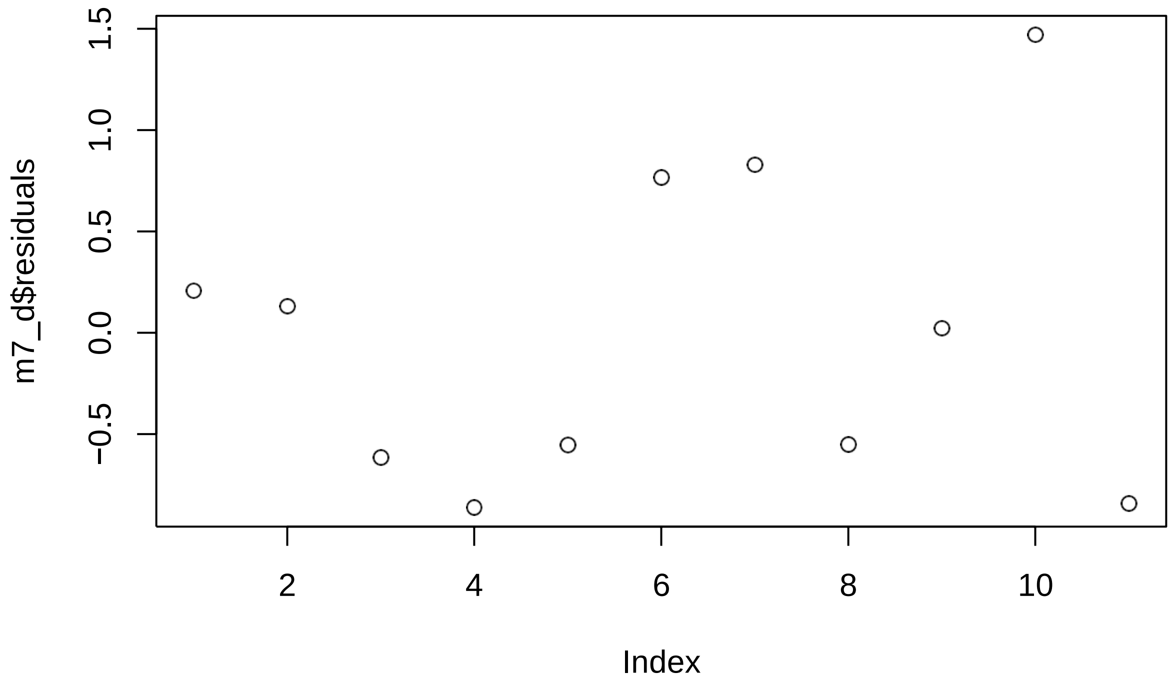


```
#same as for lm  models,  regression  improves  (std.  res.  > 3)
#after  removal  of  outlier.  Further,  regression  does  not  need
#to use a 1corrected1  copy number when 1Day1  variable included.
plot(lme)
```

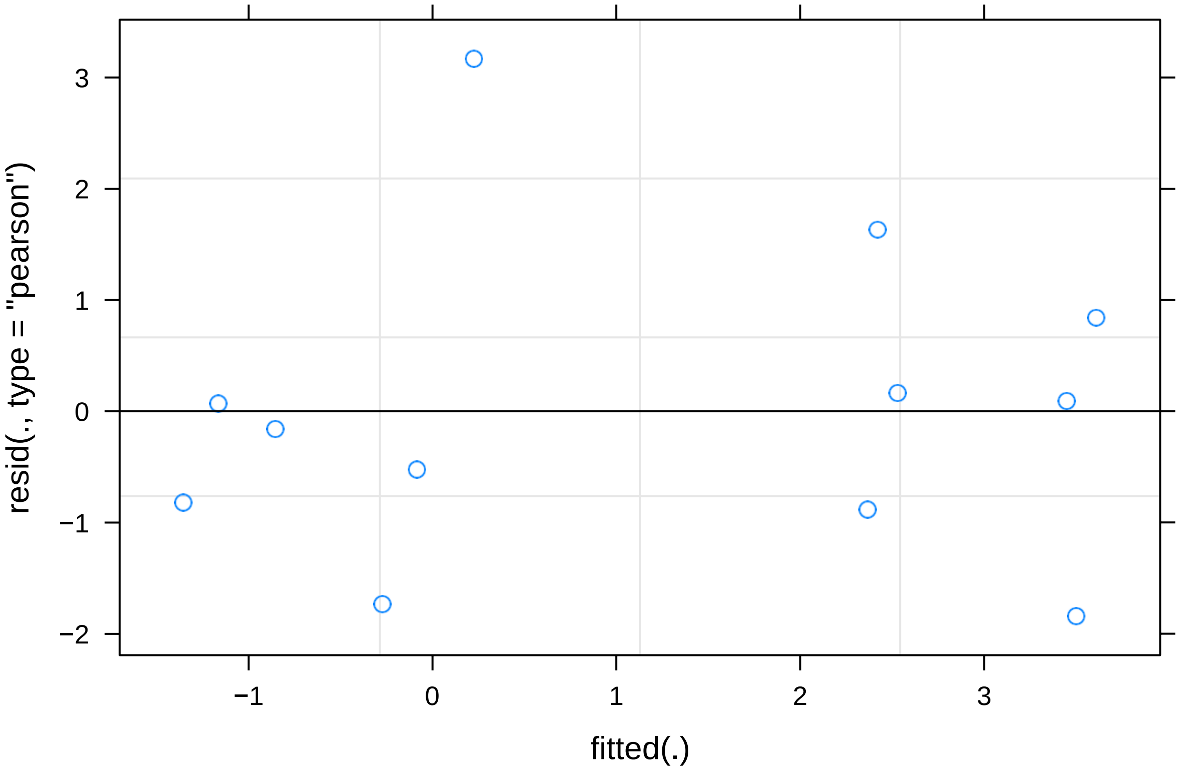


```
plot(glme)
```

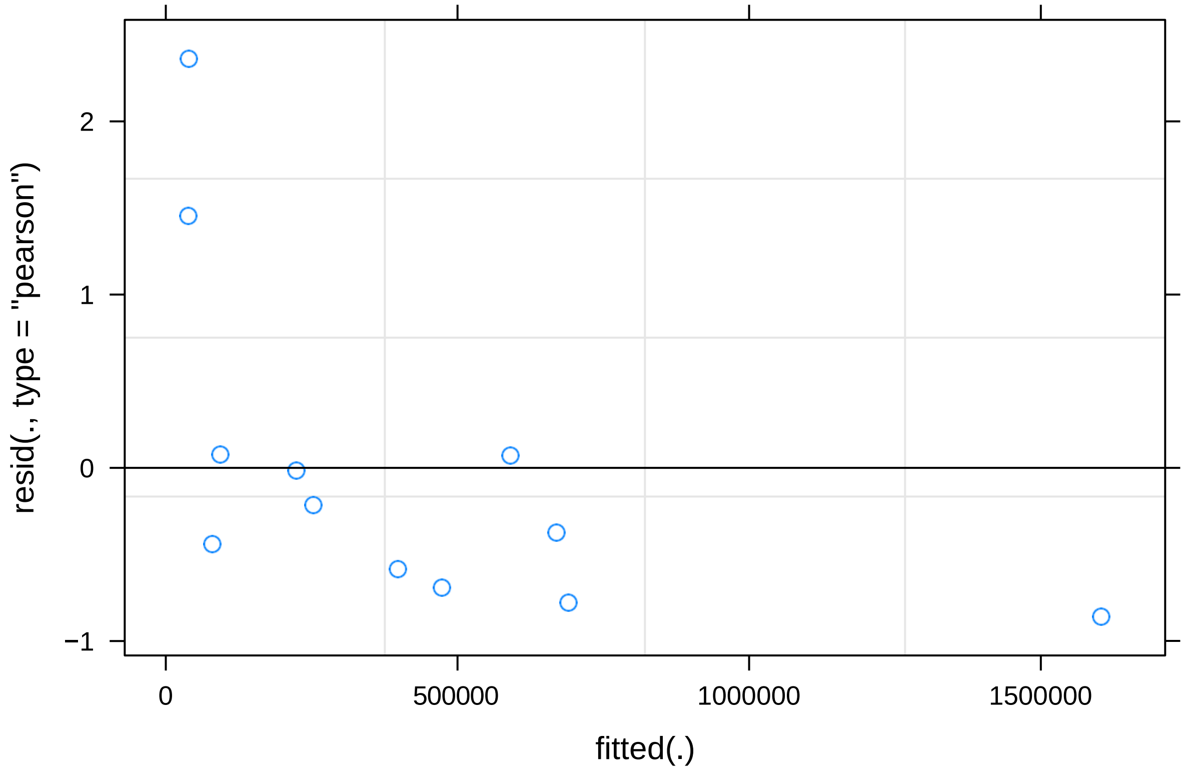


```
#lme appears to have one outlier, while glme does not. However,
#model looks to be heteroskedastic, which is not desible  for
#linear  models.
plot(glme_KD)
```

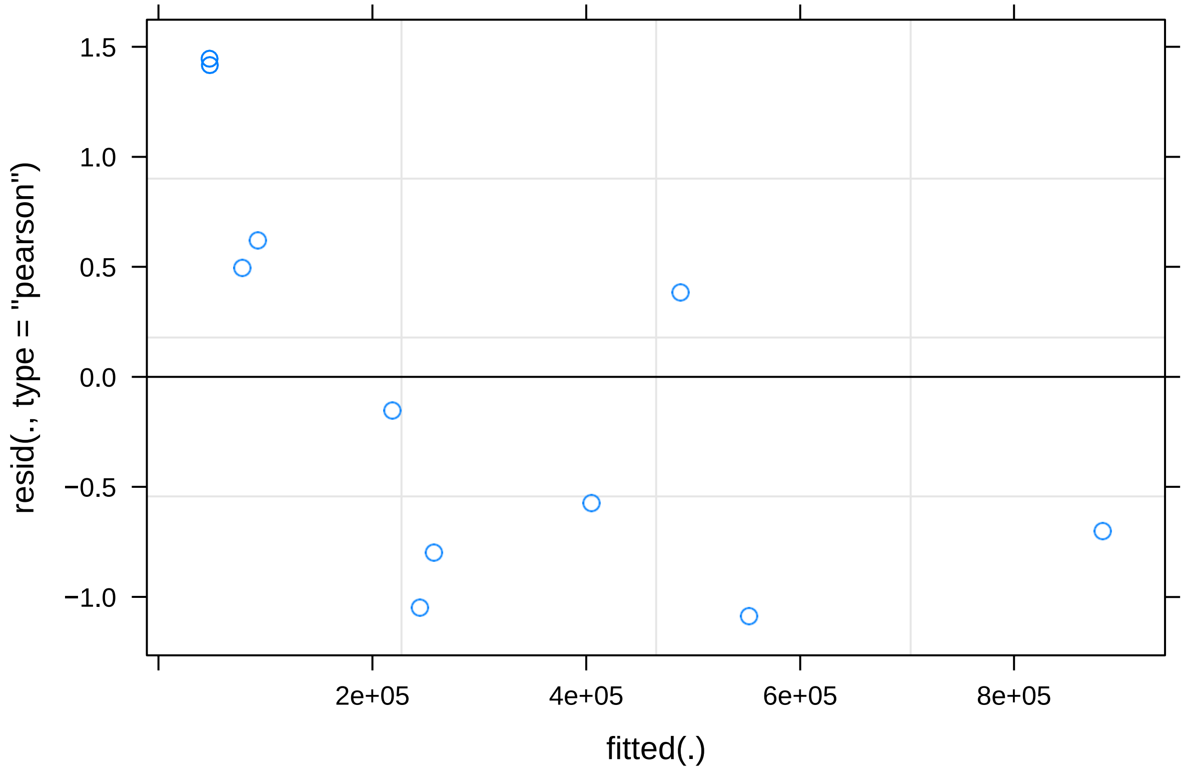


```
#looks less heteroskedastic after removing outlier, but still
#a  potential.

#CIs when removing outlier
#lm_KD
lm1ci1_KD <- exp(Confint(lm2))
lm1ci1_KD[2,]  <-  exp(-Confint(lm2)[2,])
lm1ci1_KD
```

```
##                Estimate       2.5 %       97.5 %
## (Intercept) 0.17961466  0.02539343  1.270463705
## SampleN     0.01320557  0.05979813  0.002916264
## Day         1.11129671  0.67171735  1.838541730
```

```
#lm_KD_d
lm1ci1_KDd <- exp(Confint(lm2_d))
lm1ci1_KDd[2,]  <-  exp(-Confint(lm2_d)[2,])
lm1ci1_KDd
```

```
##                Estimate       2.5 %       97.5 %
## (Intercept) 0.25176647  0.08886565  0.713283003
## SampleN     0.01279405  0.05240546  0.003123488
```

```
#lm_KD(vcovCR)
cov.m1 <- vcovCR(lm2, type="CR2", cluster = pointdata_KD$Comparison)
lm1ci_KD <- exp(Confint(lm2, vcov. = cov.m1))
```

```
## Standard errors computed by cov.m1
```

```
lm1ci_KD[2,] <- exp(-Confint(lm2, vcov. = cov.m1)[2,])

## Standard errors computed by cov.m1

lm1ci_KD

##                Estimate      2.5 %      97.5 %
## (Intercept) 0.17961466 0.10639972 0.303209691
## SampleN     0.01320557 0.01978414 0.008814489
## Day         1.11129671 0.97120300 1.271598618

#glm.nb_KD
glmci1_KD <- exp(Confint(m7))
glmci1_KD[2,]  <-  exp(-Confint(m7)[2,])
glmci1_KD

##                Estimate      2.5 %      97.5 %
## (Intercept) 0.20697138 0.06642497 0.770702235
## SampleN     0.00982711 0.02629577 0.003771916
## Day         1.12249192 0.81575913 1.558368214

#glm.nb_KD(vcovCR)
cov.m1 <- vcovCR(m7, type="CR2", cluster = copydata_wide_KD$Comparison)
glmci_KD <- exp(Confint(m7, vcov. = cov.m1))

## Standard errors computed by cov.m1

glmci_KD[2,] <- exp(-Confint(m7, vcov. = cov.m1)[2,])

## Standard errors computed by cov.m1

glmci_KD

##                Estimate      2.5 %     97.5 %
## (Intercept) 0.20697138 0.06065580 0.70623346
## SampleN     0.00982711 0.02534814 0.00380983
## Day         1.12249192 0.81848318 1.53941845

#glm.nb_KD_d
glmci1_KDd <- exp(Confint(m7_d))
glmci1_KDd[2,]  <-  exp(-Confint(m7_d)[2,])
glmci1_KDd

##                 Estimate     2.5 %      97.5 %
## (Intercept) 0.300955332 0.1595402 0.672842372
## SampleN     0.009319618 0.0251800 0.003549947
```

Validating and comparing models with 'performance'

```
compare_performance(lm1,lm1_d,m1,m4,m4_dc,lme,glme,   rank   =  TRUE)
```

```
## # Comparison of Model Performance Indices
##
## Model |     Type |      AIC |      BIC |   RMSE |  Sigma |          BF | Performance_Score
## ---------------------------------------------------------------------------------------------
## glme  | glmerMod |   341.00 |   343.42 |   0.95 |   1.00 | BF < 0.001 |            99.99%
## m4_dc |   negbin |   341.08 |   342.54 |   1.13 |   1.24 | BF < 0.001 |            99.97%
## m4    |   negbin |   459.77 |   461.71 |   1.11 |   1.29 | BF < 0.001 |            99.97%
## lme   |  lmerMod |    51.60 |    54.02 |   1.34 |   1.60 | BF = 0.548 |            99.94%
## lm1   |       lm |    50.88 |    52.82 |   1.44 |   1.67 |  BF = 1.00 |            99.94%
## lm1_d |       lm |    50.45 |    51.90 |   1.54 |   1.69 |  BF = 1.58 |            99.93%
## m1    |      glm | 2.07e+06 | 2.07e+06 | 415.35 | 479.61 | BF < 0.001 |             0.00%
##
## Model glme (of class glmerMod) performed best with an overall performance score of 99.99%.
```

```
#essentially, all models do an OK job at representing their data
#(transformed/corrected or not), except for the Poisson regression
#(probably due to overdispersion). See 1performance1  github for
#further details.




#plotting models1    performance before removal of outlier
plot(compare_performance(lm1,lm1_d,m1,m4,m4_dc,lme,glme, rank = TRUE))
```

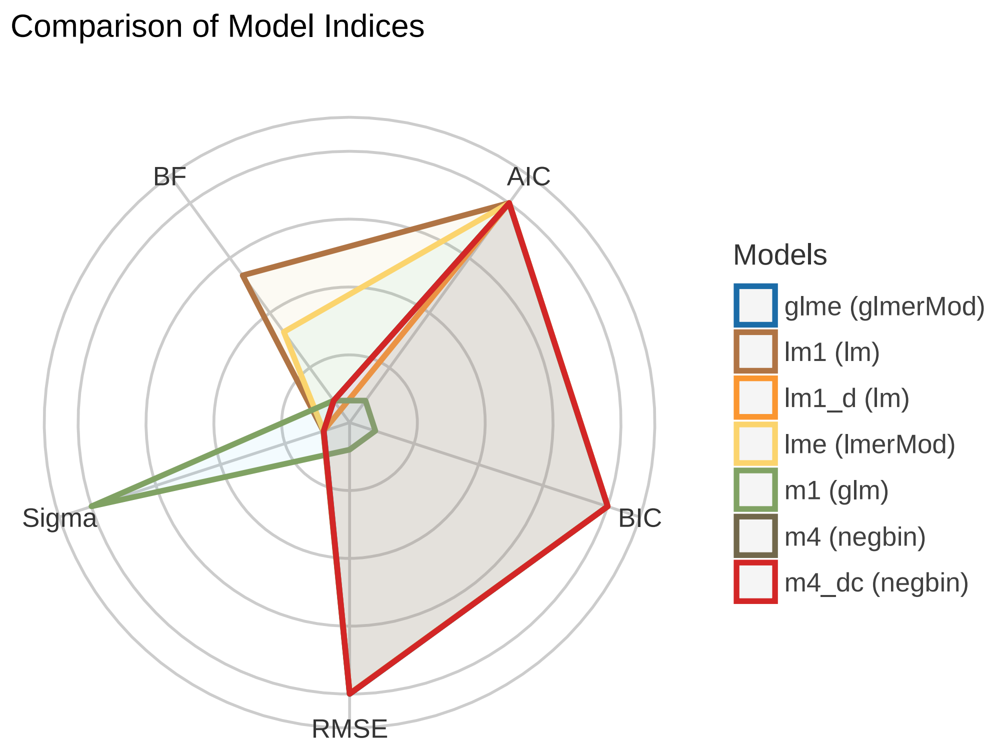

Model labels:
lm1 - linear model of log transformed copy number ratio
lm1_d - linear model of log transformed copy number ratio without 'Day' as X variable
lme - linear mixed-effect model of log transformed copy number ratio
m1 - Poisson model of copy number ratio (found to be over-dispersed)
m4 - Negative binomial model of transformed copy number ratio
m4_dc - Negative binomial model of copy number ratio without 'Day' as X variable
glme - Negative binomial mixed-effect model of copy number ratio

Analysis references

Useful Webpages

To the best of our knowledge, these are not peer reviewed, but do contain useful information.
- https://lindeloev.github.io/tests-as-linear/
- https://stats.idre.ucla.edu/r/dae/poisson-regression/
- https://stats.idre.ucla.edu/r/dae/negative-binomial-regression/
- https://rpubs.com/Shaunson26/offsetglm
- https://ourcodingclub.github.io/tutorials/mixed-models/

R packages

lme4
Douglas Bates, Martin Maechler, Ben Bolker, Steve Walker (2015). Fitting Linear Mixed-Effects Models Using lme4. Journal of Statistical Software, 67(1), 1-48. doi:10.18637/jss.v067.i01.

DHARMa
Florian Hartig (2020). DHARMa: Residual Diagnostics for Hierarchical (Multi-Level / Mixed) Regression Models. R package version 0.3.3.0. https://CRAN.R-project.org/package=DHARMa

car
John Fox and Sanford Weisberg (2019). An {R} Companion to Applied Regression, Third Edition. Thousand Oaks CA: Sage. URL: https://socialsciences.mcmaster.ca/jfox/Books/Companion/

stargazer
Hlavac, Marek (2018). stargazer: Well-Formatted Regression and Summary Statistics Tables. R package version 5.2.1. https://CRAN.R-project.org/package=stargazer

msm
Christopher H. Jackson (2011). Multi-State Models for Panel Data: The msm Package for R. Journal of Statistical Software, 38(8), 1-29. URL http://www.jstatsoft.org/v38/i08/.

ggpubr
Alboukadel Kassambara (2020). ggpubr: 'ggplot2' Based Publication Ready Plots. R package version 0.4.0. https://CRAN.R-project.org/package=ggpubr

performance
Lüdecke, Makowski, Waggoner & Patil (2020). Assessment of Regression Models Performance. CRAN. Available from https://easystats.github.io/performance/

clubSandwich
James Pustejovsky (2020). clubSandwich: Cluster-Robust (Sandwich) Variance Estimators with Small-Sample Corrections. R package version 0.5.2. https://CRAN.R-project.org/package=clubSandwich

MASS
Venables, W. N. & Ripley, B. D. (2002) Modern Applied Statistics with S. Fourth Edition. Springer, New York. ISBN 0-387-95457-0

merDeriv
Wang T, Merkle EC (2018). "merDeriv: Derivative Computations for Linear Mixed Effects Models with Application to Robust Standard Errors." *Journal of Statistical Software, Code Snippets*, *87* (1), 1-16. doi: 10.18637/jss.v087.c01 (URL: https://doi.org/10.18637/jss.v087.c01).

tidyverse
Wickham et al., (2019). Welcome to the tidyverse. Journal of Open Source Software, 4(43), 1686, https://doi.org/10.21105/joss.01686
